# Critical metabolic pathways and SAD/FADs, WRI1s, and DGATs cooperate for high-oleic acid oil production in developing oil tea (*Camellia oleifera)* seeds

**DOI:** 10.1093/hr/uhac087

**Published:** 2022-04-21

**Authors:** Jihong Yang, Beibei Chen, Sehrish Manan, Penghui Li, Chun Liu, Guangbiao She, Shancen Zhao, Jian Zhao

**Affiliations:** State Key Laboratory of Tea Plant Biology and Utilization, College of Tea and Food Science and Technology, Anhui Agricultural University, Hefei, 230036, China; National Laboratory of Crop Genetic Improvement, Huazhong Agricultural University, Wuhan, 340070, China; National Laboratory of Crop Genetic Improvement, Huazhong Agricultural University, Wuhan, 340070, China; State Key Laboratory of Tea Plant Biology and Utilization, College of Tea and Food Science and Technology, Anhui Agricultural University, Hefei, 230036, China; BGI Institute of Applied Agriculture, BGI–Shenzhen, Shenzhen 518083, China; State Key Laboratory of Tea Plant Biology and Utilization, College of Tea and Food Science and Technology, Anhui Agricultural University, Hefei, 230036, China; BGI Institute of Applied Agriculture, BGI–Shenzhen, Shenzhen 518083, China; State Key Laboratory of Tea Plant Biology and Utilization, College of Tea and Food Science and Technology, Anhui Agricultural University, Hefei, 230036, China

## Abstract

Oil tea trees produce high-quality edible oils with desirably high oleic acid (18:1) and low linoleic (18:2) and linolenic (18:3) fatty acid (FA) levels, but limited understanding of tea oil biosynthesis and regulation has become a significant obstacle for the breeding of high-yield and -quality oil tea varieties. By integrating metabolite and transcriptome analyses of developing oil tea seeds, we dissected the critical metabolic pathways, including glycolysis, fatty acid, and triacylglycerol (TAG) biosynthesis, as well as genes essential for tea seed oil production. Two plastidic stearoyl-acyl carrier protein desaturases (CoSAD1 and 2) and two endoplasmic reticulum-localized FA desaturases (CoFAD2 and 3) were functionally characterized as responsible for high 18:1 and low 18:2 and 18:3 proportions in tea oils. Two diacylglycerol *O-*acyltransferases (CoDGAT1 and 2) that may prefer to synthesize 18:1-TAG were functionally characterized and might be also important for high 18:1-TAG production. The highly expressed CoWRI1a and b were identified and characterized as activators of glycolysis and regulators of directing source carbon flux into FA biosynthesis in developing oil tea seeds. The upregulated *CoSAD*s with downregulated *CoFAD2* and *CoFAD3* at the late seed developmental stages mainly accounted for high 18:1 levels. Two CoDGATs might be responsible for assembling TAGs with oleoyl acyl chains, whilst two CoWRI1s regulated carbons from parental sources, partitioning into oil production in oil tea embryo sinks. This study provides a deep understanding of the biosynthesis of tea seed oils and information on genes that may be used as molecular markers to breed oil tea varieties with higher oil yield and quality.

## Teaser

**Significance statement** 1. Oil tea seeds displayed an increase in 18:1 level but continuous decreases in 16:0, 18:0, 18:2, and 18:3 contents over seed developmental stages until maturation.

2. The upregulated plastidic *CoSAD*s together with the downregulated ER *CoFAD2* and *CoFAD3* likely account for the high 18:1 level and low 18:2 and 18:3 levels in oil tea seeds.

3. Highly expressed *CoWRI1*s activated glycolysis and fatty acid biosynthesis for the CoDGATs-mediated ER assembly of TAGs with oleoyl acyl chains in developing oil tea seeds.

## Introduction

*Camellia oleifera* Abel., commonly known as the oil tea or oil camellia, is a broadleaf perennial. It is a major woody oilseed tree mainly cultivated in the mountainous areas of southern China, and is also widely cultivated in other countries. The total oil tea tree plantation area is ~5.3 million hectares, of which ~3.1 million hectares are located in China [1]. Oil tea seeds are used for the extraction of edible oil, also called tea oil, which is composed of monounsaturated and polyunsaturated fatty acids (FAs) and minor saturated FAs [2]. Collectively, tea oil accounts for ~25–40% of dry oil tea seeds, which is less than the proportion in other major oilseed crops, such as rapeseeds (40–50%), peanut (35–40%), or oil palm (30–50%) [3]. Therefore, there is still more room to increase the amount of oil in oil tea seeds. Moreover, ~80% of tea oils is oleic acid (18:1) and ~7% is linoleic acid (18:2), which gives what is regarded as the most suitable ratio of 18:1/18:2 for human health, comparable to that of olive oil. Oil tea seeds are also rich in saponins, vitamin E, polyphenols, phytosterols, and carotenoids [3–7]. Thus, tea oils are also used as herbal medicine for various health benefits, such as reducing serum triglycerides, improving the digestive system, reducing bad cholesterol, lowering blood pressure, increasing high-density lipoproteins, and strengthening the immune system [2, 4, 8]. Due to high levels of antioxidants, potential health benefits, and long shelf life, tea oil is recommended by the Food and Agriculture Organization (FAO) of the United Nations as a healthy vegetable oil [9, 10]. In addition, tea oil is widely used in the cosmetics industry, to prepare margarines, lubricants, and rustproof oils because of its special physiochemical properties [2, 3]. Therefore more tea oil production with enhanced components is the current demand of tea oil breeders and consumers as well [11, 12].

Given the increasing market demand for high-quality tea oils and the huge vegetable oil supply deficit in China, the oil tea cultivation acreage has increased drastically in recent years. However, still only ~3.4 million hectares of oil tea trees are cultivated in China, producing <2.3 million tons of tea seed oil annually, accounting for only 1.2% of the total consumption of edible oils in China [1, 13]. The government of China has planned to double the oil tea tree planting area (6.8 million hectares) and increase the overall production of tea seed oil to 4.6 million tons to meet the challenge of increased demand for supplies of edible oils [1, 13]. Thus, FA biosynthesis and triacylglycerol (TAG) production in developing oil tea seeds must be understood much better to facilitate the breeding of *C. oleifera* varieties with high oil yield.

Several studies on transcriptome profiling of developing oil tea seeds have been conducted [1, 14–16]. These preliminary transcriptome studies have generally described pathway gene transcripts and revealed the evolution of metabolic genes [1, 14], by focusing on one or two metabolic genes without functional characterization [14], comparing the transcriptomes of high- and low-oil tea seeds to characterize differential gene expression [15], or combining proteomic and transcriptomic analyses to reveal key metabolic genes or enzymes for oil tea seed quality [16], as well as more recent genome sequencing of oil tea trees [17, 18]. However, none of these studies has functionally characterized any of the key oil synthesis genes to support hypotheses. Many fundamental questions remain unanswered, such as what genetic factors primarily determine the diversity of oil yield or composition in oil tea seeds among the numerous available varieties. Despite the 2000-year history of oil tea tree cultivation and tea seed oil utilization, extensive studies are needed to understand these quantitative traits and quality parameters of oil tea trees and tea seed oils. Here, metabolite profiling and transcriptome analysis of developing oil tea seeds at various developmental stages were performed to identify critical metabolic pathways and genes for the production of high-oleic-acid tea seed oils. We functionally characterized several genes involved in metabolic pathways from glycolysis, FA synthesis, to TAG assembly, including two stearoyl-acyl carrier protein (ACP) desaturases (CoSAD1 and 2), two FA desaturases (CoFAD2 and 3), and two diacylglycerol acyltransferases (CoDGAT1 and 2). Two oil tea WINKLED1 homologs (CoWRIs) were also characterized as oil biosynthesis regulators for oil production. Our study further employed transcriptome and FA profiling of developing seeds from more than 14 local *C. oleifera* varieties and verified the close correlation between the oil compositions and contents in *C. oleifera* seeds with expression levels of these genes.

## Results

### Changes in storage substances in oil tea seeds at various development stages

**Figure 1:**
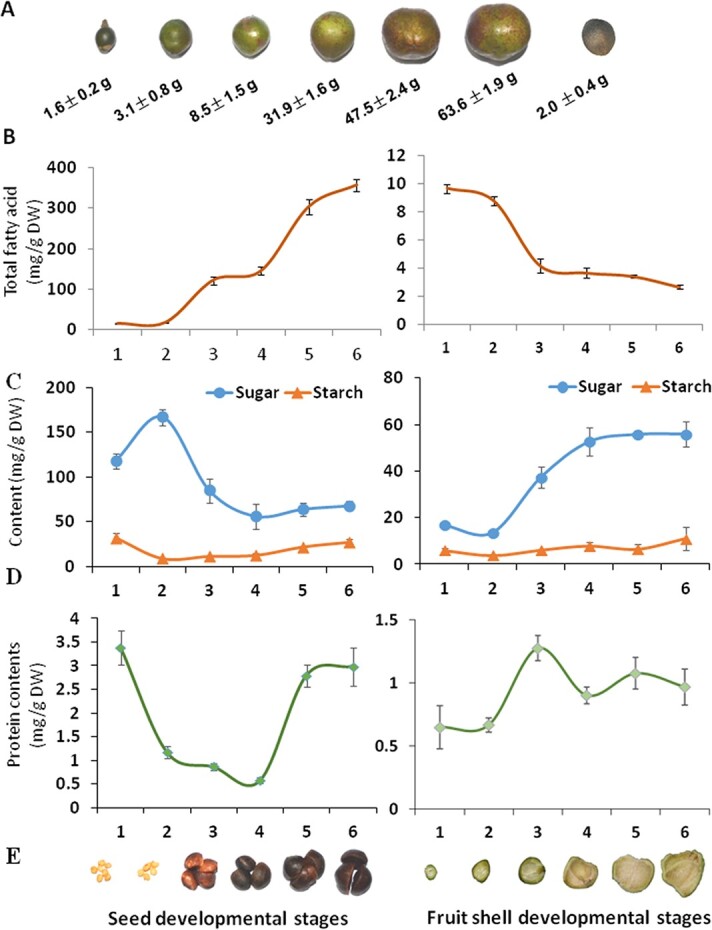
Analyses of seed storage metabolites in developing oil tea seeds. (A) Oil tea fruits at various developmental stages (first six from left) and dry fruit (rightmost). The cultivar ‘Changlin #4’ is used as a representative to show developmental stages. The weight of fruit is given below each fruit. (B) FA contents in developing oil tea seeds (left panel) and fruit shells (right panel). (C) Starch and sugar contents in developing oil tea seeds (left panel) and fruit shells (right panel). (D) Protein contents in developing oil tea seeds (left) and fruit shells (right). (E) Appearance of seeds (left panel) and fruit shells (right panel) at various developmental stages. Data represent three independent experiments and are shown as means ± standard deviation (*n* = 3). Variance between numbers at various developmental stages was analyzed.

We profiled and measured mature *C. oleifera* seed oil in comparison with other three common edible oils: peanut oil, rapeseed oil, and olive oil. Tea seed oil consisted of 6% palmitic acid (16:0), 1% stearic acid (18:0), 79% 18:1, 7% 18:2, and 0.3% linolenic acid (18:3). Thus, the FA composition of tea seed oil is similar to that of olive oil, but has much fewer polyunsaturated FAs (18:2 and 18:3) than rapeseed and soybean oils [14]. In total, the dry mass of the mature seeds of the ‘Changlin #4’ cultivar contains up to 35% oil (Fig. 1). To understand how these major valuable nutrients and basic energy substances, such as proteins and carbohydrates, are synthesized during the seed developmental stages, we first measured different types of nutrient substances in both seeds and fruit shells of a local oil tea tree cultivar (*C. oleifera* ‘Changlin #4’) at six developmental stages (Fig. 1A). The total FA contents in oil tea seeds at six development stages changed significantly from 2.2 at Stage 1 to 155.5 mg/g at Stage 6, and we observed a slight increase in the early period (Stage 1–Stage 4) and a considerable increase from Stage 5 to Stage 6 (Fig. 1B). We sought to understand how carbohydrates derived from leaf photosynthesis and transported via phloem into the seeds are dynamically metabolized and partitioned into oils, proteins, and starches in oil tea seeds. For this, the accumulation patterns of seed storage substances such as starches, soluble sugars, and proteins at six developmental stages of oil tea seeds and fruit shells were measured (Fig. 1C). In developing seeds, the starch content decreased from 31.9 to 9.4 mg/g from Stage 1 to Stage 2 and then increased to 27.0 mg/g at Stage 6, and soluble sugar content increased from 117.7 to 166.7 mg/g from Stage 1 to Stage2 and then decreased to 67.8 mg/g at Stage 6 during the ripening process, suggesting partitioning of these carbohydrates into FAs and proteins in seed development (Fig. 1C). The protein contents of developing oil tea seeds decreased at Stage 4 and then increased to 2.9 mg/g at Stage 6 (Fig. 1D). The increasing oil contents suggested a primary partitioning of these carbohydrates into the FA synthesis process during seed development. In fruit shells, soluble starch was slightly decreased. Soluble sugar continuously increased and was maintained at levels from 16.7 to 55.9 mg/g during the ripening process. Although the proteins of fruit shells remained similar at all developmental stages, the total FA contents in fruit shells were relatively low and decreased continuously in a manner opposite to the trend observed in the developing seeds (Fig. 1).

### *De novo* assembly and annotation of the developing oil tea seed transcriptome

To dissect the biosynthesis of these seed storage substances, an RNA-Seq analysis of oil tea seeds of *C. oleifera* cv. ‘Changlin #4’ was conducted. RNA-Seq generated a range of 6–8 G clean reads for each sample, which were assembled and annotated into 104 287 transcripts assigned to biological processes, 97 270 transcripts assigned to cellular components, and 52 691 transcripts assigned to molecular functions (Supplementary Data Fig. S1). Approximately 29.25% of the entire homologous sequences had significant matches with genes from *Vitis vinifera*, followed by *Coffea canephora* (5.32%), *Sesamum indicum* (4.98%), and *Theobroma cacao* (4.84%). More than 63% were matched to the tea plant genome sequence of *Camellia sinensis* var. *sinensis* (http://tpia.teaplant.org/download.html).

**Figure 2:**
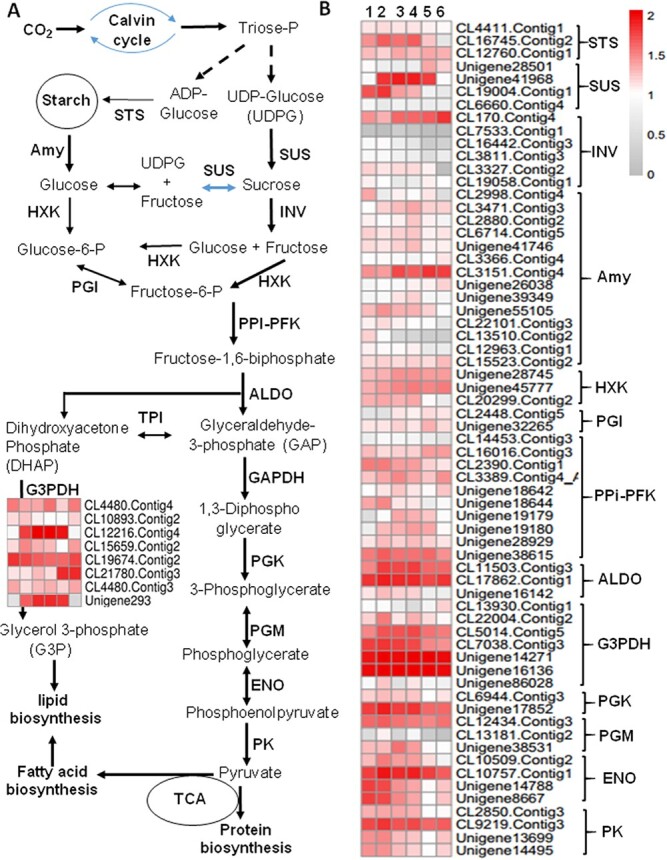
Expression patterns of starch and sucrose metabolism and glycolysis pathway genes in developing oil tea seeds. (A) Schematic of the starch and sucrose metabolism and glycolysis pathways and corresponding genes involved in the pathways. (B) Heat map showing expression patterns of genes involved in the metabolic pathways of developing oil tea seeds. (C) Heat map of genes involved in lipid metabolism. (D) qRT–PCR verification of expression patterns of the major genes involved in starch and sucrose synthesis and glycolysis pathways. All qRT–PCR data are expressed as means ± standard deviation (*n* = 3) from three independent experiments. STS, starch synthase; SUS, sucrose synthase; INVs, invertases; HXK, hexokinase; PGI, glucose phosphate isomerase; PPi-PFK, phosphofructokinase; ALDO, fructose-bisphosphate aldolase; GAPDH, glyceraldehyde-3-phosphate dehydrogenase; G3PDH, glycerol 3-phosphate dehydrogenase; GK, phosphoglycerate kinase; PGM, 2,3-bisphosphoglycerate-independent phosphoglycerate mutase; ENO, enolase; PK, pyruvate kinase; Amy, amylase; TPI, triose phosphate isomerase; SPS, sucrose phosphate synthase.

**Figure 2:**
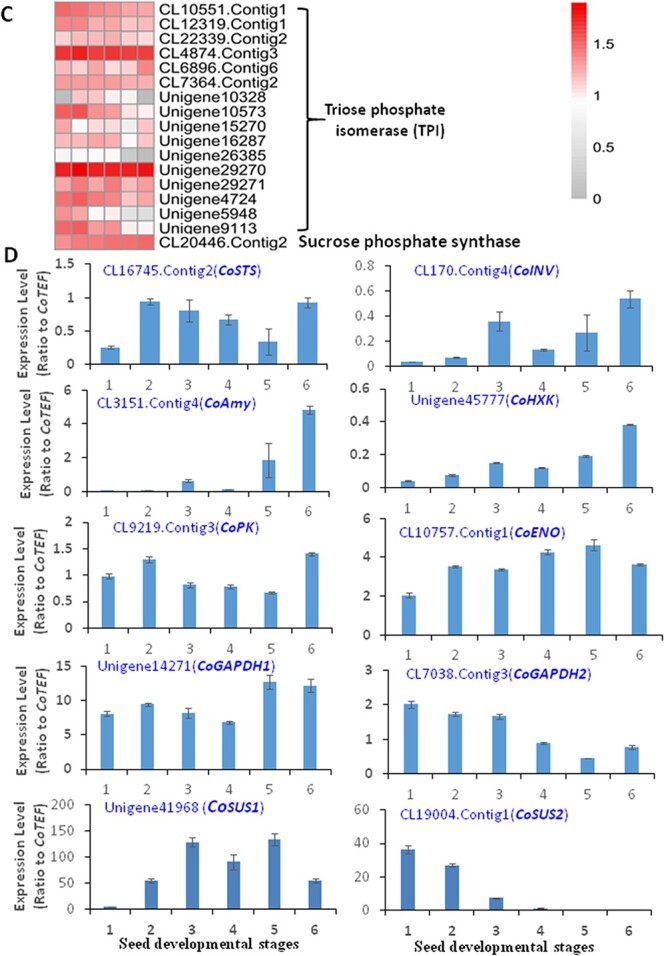
Continued

### Changes in glycolysis pathway genes in developing oil tea seeds

Starch is an important storage form of carbohydrates in many seeds. Although starch accounted for a lower portion of storage substances compared with TAGs, the synthesis and degradation of starch in oil tea seeds is closely related to sucrose metabolism and carbon partitioning from the sources [19, 20]. The glycolysis pathway is the major pathway involved in the oxidation of glucose to pyruvate for either FA or amino acid synthesis in all developing seeds. Using the Kyoto Encyclopedia of Genes and Genomes (KEGG), these metabolic pathways and corresponding metabolic genes were obtained from transcriptome data of developing oil tea seeds (Supplementary Dataset S1). Three genes, *CL4411.Contig1*, *CL16745.Contig2*, and *CL12760.Contig1*, encoded granule-bound starch synthases (CoSTS), which catalyze the transfer of glucose from ADP-glucose to glucose-containing polysaccharides in 1,4-α-linkages (Fig. 2). These genes were upregulated in different manners during seed development. Among 14 chloroplastic/amyloplastic amylase (Amy) genes, *CL3151.Contig4* is the main transcript upregulated in oil tea seeds from the seed filling stage to the maturation stage. Both invertase (INV) and sucrose synthase (SUS) catalyze the sucrose cleavage reaction in alfalfa plants [21–24]. However, a recent study distinguished the functions of INV and SUS genes in plants [23]. SUS is a cytoplasmic glycosyl transferase catalyzing the reversible UDP-dependent cleavage of sucrose into UDP-glucose and fructose. INV irreversibly cleaves sucrose into glucose and fructose in equal proportions, which are further phosphorylated by hexokinase (HXK) into their 6-phosphate forms. Then, glucose-6-phosphate is converted into fructose-6-phosphate, fructose-1,6-diphosphate, and glyceraldehyde-3-phosphate by glucose-6-phosphate isomerase (GPI), phosphofructokinase 1 (PFK1), and fructose-bisphosphate aldolase (ALDO), respectively. The *CoINV* transcript *CL170.Contig4* steadily increased to the highest expression level at the late developmental stage. In contrast, five other weakly expressed *CoINV* transcripts, comprising *CL7533.Contig1*, *CL16442.Contig3*, *CL3811.Contig3*, *CL3327.Contig2*, and *CL19058.Contig1*, were markedly downregulated at late seed developmental stages (Fig. 2). Among the four genes encoding SUSs, two *CoSUS* genes exhibited much higher expression levels. *CL19004.Contig1* displayed the highest expression level in embryogenesis and early developmental stages (Stages 1 and 2), whereas *Unigene41968* maintained the highest expression level during the early stage to seed filling (from Stage 2 to 4). Then, the levels of these transcripts decreased until seed maturation (Supplementary Dataset S1).

The transcript levels of most *CoHXK* genes, such as *Unigene28745* and *Unigene45777*, are increased. However, *CL20299.Contig2* slightly increased and then decreased at the late developmental stage. Overall, the levels of two *CoGPI* genes, *CL2448.Contig5* and *Unigene32265*, were increased at all developmental stages, which is consistent with the increase in oil content throughout seed development. The highly expressed *CoPFK1* genes *CL2390.Contig1*, *Unigene18644*, and *Unigene38615* peaked in the embryogenesis and early seed developmental stages and then decreased significantly during seed maturation. The levels of *CL16016.Contig3*, *CL3389.Contig4*, *Unigene38615*, *Unigene18642*, *Unigene19179*, *Unigene19180*, and *Unigene28929* transcripts were high in early stages and subsequently decreased during the maturation phase. Glyceraldehyde -3-phosphate (GAP) is one of the most important metabolites in glycolysis, and most *CoALDO* genes, such as *CL11503.Contig3* and *CL17862.Contig1*, were highly expressed at the early developmental stages. As a key catalyzing enzyme, glyceraldehyde 3-phosphate dehydrogenase (GAPDH) is encoded by multiple genes, and most of these genes, including *CL5014.Contig5*, *CL7038.Contig3*, *Unigene14271*, and *Unigene16136*, were highly expressed at early seed developmental stages (Fig. 2). Other downstream glycolytic genes, including the genes encoding phosphoglycerate kinase (*CoPGK*, *CL6944.Contig3*, and *Unigene17852*), phosphoglycerate mutase (*CoPGM*, *CL12434.Contig3*, *CL13181.Contig2*, and *Unigene38531*), phosphopyruvate hydratase (enolase, *CoENO*), major isogenes (*CL10509.Contig2*, *CL10757.Contig1*, *Unigene14788*, and *Unigene8667*), and pyruvate kinase (*CoPK*, major isoforms *CL2850.Contig3*, *CL9219.Contig3*, *Unigene13699*, and *Unigene14495*), generally exhibited decreasing trends with different characteristics during seed maturation. Several highly expressed triose phosphate isomerases (TPIs), which catalyze the reversible conversion of dihydroxyacetone phosphate (DHAP) and GAP, were also identified. Their transcripts, including *CL4874.Contig3*, *Unigene29270*, *Unigene10573*, and *Unigene4724*, were maintained at high levels during all seed developmental stages (Fig. 2C). Glycerol 3-phosphate dehydrogenase (G3PDH) further catalyzes dihydroxyacetone phosphate to glycerol 3-phosphate (G3P), which acts as a universal skeleton precursor for glycerolipid synthesis [25]. The sucrose phosphate phosphatase gene, *CL20446.Contig2*, was only active at the onset of maturation stage (Fig. 2C).

RNA-Seq results were validated by qRT–PCR analysis of 10 random candidate genes (Fig. 2D): *CsSTS* (*CL16745.Contig2*), *CoINV* (*CL170.Contig4*), *CoAmy* (*CL3151.Contig4*), *CoHXK* (*Unigene45777*), *CoPK* (*CL9219.Contig3*), *CoENO* (*CL10757.Contig1*), *CoGAPDH* (*Unigene14271* and *CL7038.Contig3*), and *CoSUS* (*Unigene41968* and *CL19004.Contig1*) (Fig. 2D). We demonstrated that the expression profiles of these 10 genes were mostly consistent between RT–PCR and RNA-Seq experiments; thus, the expression profiles of transcriptome results had high confidence (Fig. 2D).

### Expression of fatty acid and triacylglycerol synthesis genes in developing oil seeds

The major FA in oil tea seeds is 18:1, and the 18:1 FA contents exhibited the greatest increase at all oil tea seed developmental stages. The major increase in oils occurred at Stages 5 and 6. The proportions of 16:0, 18:0; 18:2, and 18:3 FAs increased (Fig. 3A). Stages 4–6 represent the seed developmental period when late seed development and seed filling actively occurred (Fig. 3A). During seed development, 18:1 TAGs increased continuously, whereas the proportions of 16:0, 18:0, 18:2, and 18:3 TAGs were reduced gradually (Fig. 3B). Regarding FA synthesis in the chloroplast, acetyl CoA carboxylase (ACCase) is the rate-limiting enzyme and converts acetyl-CoA to malonyl-CoA in the first step [26]. Among 49 ACCase transcripts that exhibited significantly different expression patterns at six developmental stages, *CL1061.Contig4*, *CL5954.Contig3*, and *Unigene6929* were highly expressed ACCase genes (Fig. 3C; Supplementary Data Fig. S2). *CL1061.Contig4*, *Unigene17024*, *Unigene6929*, and *Unigene72627* transcripts were increased in early developing seeds until Stage 4 and then decreased during seed maturation (Fig. 3C; Supplementary Dataset S1). However, *CL2005.Contig8* and *CL5954-Contig3* transcripts steadily increased in most developmental stages, peaked at Stage 5, and then decreased (Fig. 3C; Supplementary Dataset S1). The FA elongation cycle is catalyzed by the FA synthase complex. 3-Ketoacyl-ACP synthase (KAS III), β-ketoacyl synthetase (KASI), β-ketoacyl-acyl-carrier-protein (ACP) synthase (KAS III); β-ketoacyl-ACP reductase (KAR), 3-hydroxyacyl-ACP dehydratase (HAD), and enoyl-ACP reductase (EAR) catalyze the successive biosynthesis of palmitoyl-ACP from malonyl-ACP (Fig. 3C; Supplementary Data Figs S3 and S4). The oil tea β-ketoacyl-ACP synthase (*CoKASII*) gene *CL1914.Contig2* as well as *CoKASIII* genes *CL1668.Contig2*, *Unigene25219*, and *Unigene25220* were upregulated at the early seed developmental Stages 1–4 and then decreased afterward at Stages 5 and 6 (Fig. 3C; Supplementary Data Fig. S3). Acyl-acyl carrier protein thioesterase (FATB) also plays an essential role in FA biosynthesis. As one of the major FATB genes in seeds, *CL53.Contig4* (*Unigene16664*) was significantly upregulated at early developmental Stages 1 and 2 and decreased later on. Other acyl-ACP thioesterase (FAT) genes, such as *CL7284.Contig2*, also displayed the same expression patterns in developing seeds (Fig. 3C; Supplementary Data Fig. S4; Supplementary Dataset S1).

**Figure 3:**
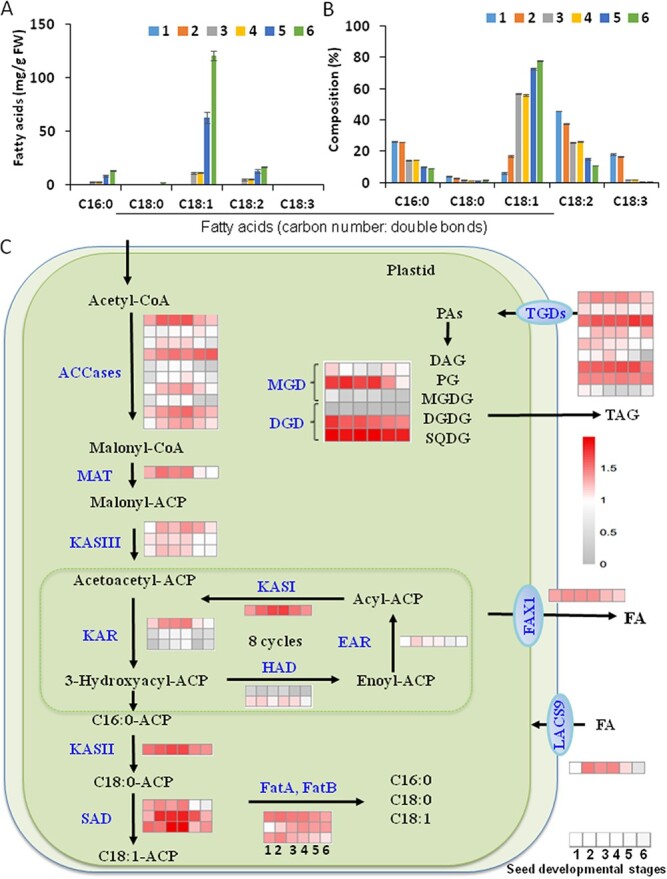
FA and TAG accumulation and expression of genes responsible for FA and TAG biosynthesis in developing oil tea seeds. (A, B) Total FA contents (A) and components (B) in TAGs of oil tea seeds at various developmental stages. (C) Heat map analysis of expression patterns of genes involved in plastidic FA biosynthesis. (D) Heat map analysis of expression patterns of genes involved in phospholipid and long-chain FA synthesis in the ER. Data represent three independent experiments and shown as means ± standard deviation (*n* = 3). Variance between numbers at various developmental stages was analyzed. Transcript IDs for the heat map column are listed in the corresponding Supplementary Data. Seed stages are divided into six stages: Stages 1, 2 …, and 6. Heat maps were generated using transcriptome data with the MeV program.

**Figure 3:**
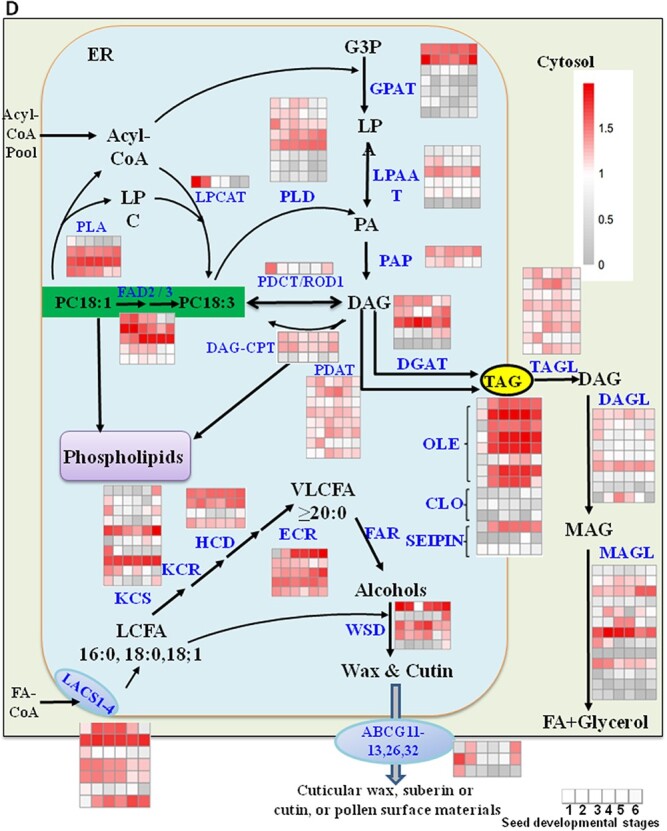
Continued

The biosynthesis of photosynthetic-specific galactolipids in the chloroplast, including monogalactosyldiacylglycerol (MGDG), digalactosyldiacylglycerol (DGDG), and sulfoquinovosyldiacylglycerol (SQDG), essentially required MGDG synthase (MGD) and DGDG synthase (DGD) (Supplementary Data Fig. S5). [11] Highly expressed transcripts of these galactolipids linked with TAG production were also found in this study [27] (Fig. 3C). G3P is transported from the cytosol into the endoplasmic reticulum (ER) and acts as an acceptor of fatty acyl-CoAs for glycerolipid synthesis, and FAs are transported into the ER by ABCA transporters potentially with the help of long-chain acyl-CoA synthetases (LACSs). [12] Positioned at the cross point in the metabolic pathways involved in the synthesis of both phospholipids and TAGs, phosphatidic acid (PA) is regarded as an important precursor. PA is hydrolyzed by PA hydrolase (PAH) into diacylglycerol (DAG), which is directly used for *de novo* TAG biosynthesis via the Kennedy pathway under the action of various acyltransferases, such as diacylglycerol:acyl-CoA acyltransferase (DGAT) and phospholipid:diacylglycerol acyltransferase (PDAT) (Fig. 3D; Supplementary Data Fig. S12). PA and DAG are also central metabolites for phospholipid synthesis, which occurs in parallel with TAG biosynthesis in the ER. Both PDAT and DGAT transfer the acyl group from phospholipids and acyl-CoAs, respectively, to DAG as a substrate to synthesize TAG. These genes were highly expressed in developing oil tea seeds; for example, the expression levels of *CoDGAT1* (*CL21487.Contig7*) were increased by ~2-fold at the late developmental Stages 5–6. *CoDGAT2-1* (*CL1666.Contig2*) displayed increased expression levels at Stages 1–4, and then levels decreased. *CoDGAT2-2* (*Unigene7038*) showed the highest expression at Stages 5–6. By comparison, *CoPDAT1* (*Unigene18970*) and *CoPDAT2* (*Unigene6465*) were expressed at lower levels in developing oil tea seeds compared with *CoDGATs* (Fig. 3D; Supplementary Data Fig. S12). Phospholipase D (PLD) and A (PLA) genes were also highly expressed in the developing seeds (Fig. 3D; Supplementary Data Figs S6–S10), and these PLDs modify phospholipids and seed storage TAGs and DAGs by affecting PA intermediate pools and acyl editing [27–29]. TAG can be successively hydrolyzed to FAs and glycerol by various lipases, such as those encoded by *TAGL*s (*CL22819.Contig2*, *CL4510.Contig1*, *Unigene27904*, and *CL806.Contig7*), *DAGL*s (*CL11004.Contig2*, *CL14845.Contig3*, and *CL4510.Contig1*), and *MAGL*s (*CL14089.Contig2*) (Fig. 3D; Supplementary Data Figs S11–S13).

Several transporter genes, such as FA transporters (FAXs), LACSs, ATP-binding cassette type G transporter (ABCGs), and triglactosyldiacylglycerol transporters (TGDs) associated either with lipid precursor inter-organelle transport or their extracellular movement, were found to be highly expressed in the current study, indicating active FA movement and exchange among the chloroplast, cytosol, and ER. The genes involved in the biosynthesis of very long chain fatty acids (VLCFAs) and waxes, including the genes encoding ketoacyl-CoA synthase (KCS), hydroxyacyl-CoA dehydrase (HAD), ketoacyl-CoA reductase (KCR), enoyl-CoA reductase (ECR), and wax ester synthase (WSD), were found to be highly expressed in developing seeds (Fig. 3D; Supplementary Data Figs S14–S17). In plants TAG is stored in special oil bodies surrounded by specific membrane proteins, including oleosins (OLEs), calcium-binding caleosins (CLOs), and sterol-binding dehydrogenases [steroleosins (SLOs)]. These proteins are abundant in seeds and significantly affect the assembly and stability of oil bodies and oil yield [11]. Based on RNA-Seq data of developing oil tea seeds, more than 30 highly expressed transcripts encoded OLEs, such as *CL13885.Contig1*, *Unigene38830*, *Unigene25677*, *CL11045.Contig2*, *CL14158.Contig1*, and *Unigene13140* (Fig. 3D; Supplementary Data Fig. S14, Supplementary Dataset S2). The expression levels of these genes were significantly upregulated during seed maturation; however, CLO-coding genes *Unigene35942*, *CL16257.Contig2*, and *Unigene44817* were present at low levels. The ER-lipid droplet protein seipin is a homo-oligomeric integral membrane protein in the ER that concentrates at junctions with cytoplasmic lipid droplets (Fig. 3D; Supplementary Data Fig. S14, Supplementary Dataset S2). Seipin-coding genes *CL15209.Contig1* and *CL22595.Contig1* were upregulated at early developmental stages and *CL21816.Contig3* was downregulated during seed development.

### Genes involved in high oleic acid accumulation in developing oil tea seeds

Generally, the high proportion of 18:1 in total FAs of oilseeds is mainly due to high activity of stearoyl-ACP desaturase (SAD) in the chloroplast and/or rather lower FA desaturase (FAD) activity in the ER [11, 30]. The *CoSAD* genes include major transcripts *CL23116.Contig1*, *CL9407.Contig 1*, and *CL17700.Contig2*, which are named *CoSAD1*, *CoSAD2*, and *CoSAD3*, respectively.

**Figure 4:**
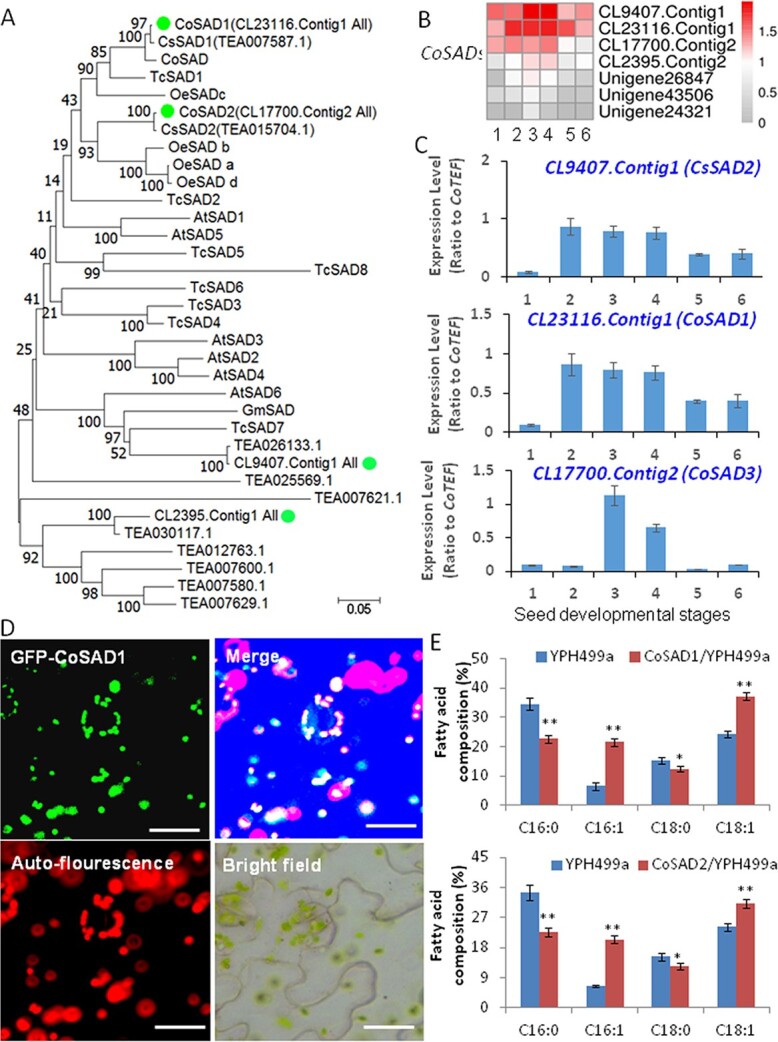
Functional characterization of *CoFAD* genes from developing oil tea seeds. (A) Phylogenetic analysis of several *CoFADs* from seed transcriptome data. (B) Heat map analyses of the expression profiles of these *CoFAD*s in developing oil tea seeds. (C) qRT–PCR verification of *CoFAD2*, *CoFAD3*, and *CoSAD7a* expression profiles in developing oil tea seeds. (D) Subcellular localization of GFP-CoSAD1 in tobacco epidermal cells. Left top panel, green fluorescence image of GFP-CoSAD1 in plastids; left bottom panel, chlorophyll autofluorescence image in chloroplasts; right top panel, merged image. Scale bars = 50 μm. (E) Increased unsaturated FAs in yeast cells when expressing *CoSAD1* (top panel) and *CoSAD2* (bottom panel), as compared with wild-type yeast cells expressing empty vector. Heat maps were generated using transcriptome data with the MeV program. Data represent three independent experiments and shown as means ± standard deviation (*n* = 3). Variance between numbers at various developmental stages was analyzed. Significance differences between nodule and root are shown as ^*^P < .05; ^**^P < .01 (Student’s *t*-test).

**Figure 5:**
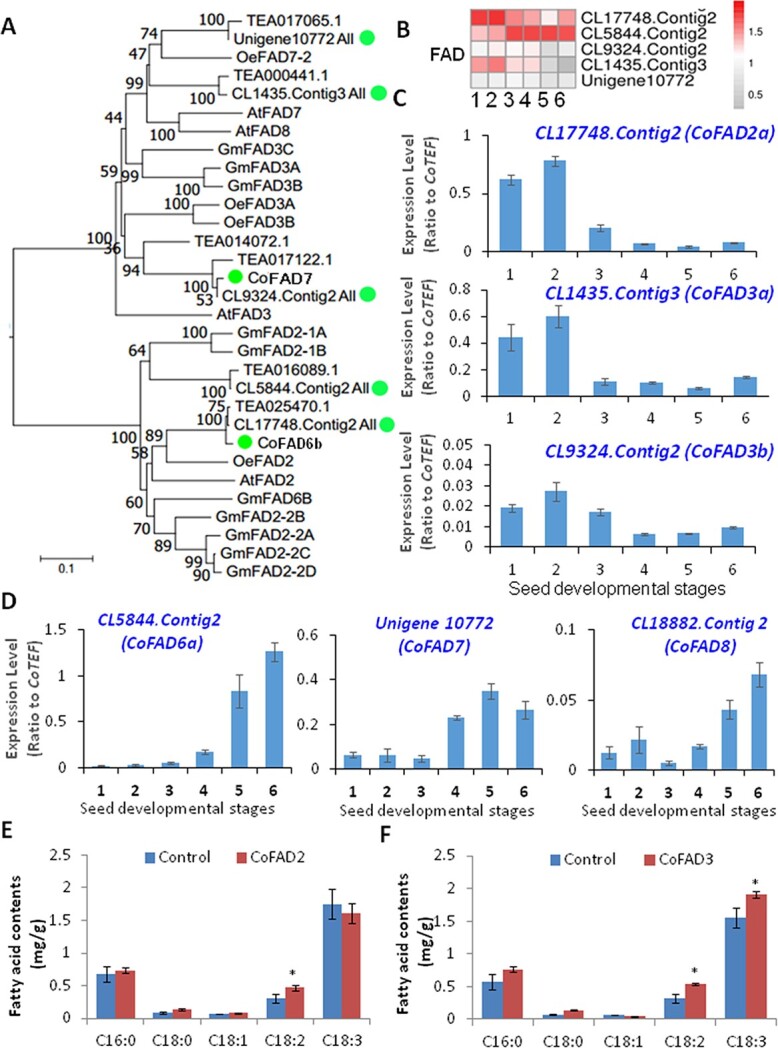
Characterization of *CoFAD* genes from developing oil *Camellia* seeds. (A) Phylogenetic analysis of several CoFADs from seed transcriptome data. (B) Heat map analyses of the expression profiles of these *CoFAD*s in developing oil *Camellia* seeds. (C) qRT–PCR verification of *CoFAD2*, *CoFAD3*, and *CoSAD7a* expression profiles in developing oil *Camellia* seeds. (D) qRT–PCR verification of *CoFAD7b*, *CoFAD6a*, and *CoSAD8* expression profiles in developing oil seeds. (E) Increased 18:2 contents in tobacco leaves when infiltrated and expressing *CoFAD2* as compared with control tobacco leaves expressing empty vector P19. (F) Increased 18:3 contents in tobacco leaves when infiltrated and expressing *CoFAD3* as compared with control tobacco leaves expressing empty vector P19. Heat maps were generated using transcriptome data with the MeV program. Data represent three independent experiments and are shown as means ± standard deviation (*n* = 3). Variance between numbers at various developmental stages was analyzed. Significance differences between nodule and root are shown as ^*^P < .05 (Student’s *t*-test).

The CoSAD sequences showed resemblance to SADs from various other oilseed species (Fig. 4). The transcripts of all *CoSAD*s were found in abundance at all stages, hence a greater amount of 18:1 at each developing stage can be correlated with high expression of *CoSAD*s*.* When CoSAD1-green fluorescence protein (GFP) fusion construct was infiltrated into tobacco epidermal cells, the CoSAD-GFP fusion signals were co-localized with chlorophyll autofluorescence, indicating their chloroplast localization (Fig. 4). To determine whether these enzymes are functionally active, we then overexpressed *CoSAD1* and *CoSAD2* in yeast cells that accumulated high levels of 16:0 and 18:0 FAs. We observed that increased levels of 16:1 and 18:1 FAs in *CoSAD1*/*2*-expressing yeast cells compared with the vector control, indicating that CoSADs indeed catalyzed 18:1 formation from 18:0-ACP or even non-specifically catalyzed 16:1 formation from 16:0-ACP (Fig. 4). The chloroplast-derived FAs were transported into the cytosol by FAXs, which constitute a large gene family in *C. oleifera*. In addition, chloroplast-specific CoFADs, including CoFAD6, 7, and 8, convert galactolipids with the C18:1 acyl chain into the corresponding galactolipids with C18:2 or C18:3 acyl chains. [31] The ER-specific CoFADs, including FAD2 and 3, catalyze the desaturation of the fatty acyl chains in phosphatidylcholines (PCs), and their genes were also detected in the transcriptome of the developing oil tea seeds. All plastidic or ER-located CoFADs were clustered with their homologs from other plants (Fig. 5A) and may exhibit different functions of modification of fatty acyl chains in PCs and membrane glycerolipids.

The transcripts of two highly expressed genes in the developing seeds, namely *CoFAD2* (CL17748.Contig2) and *CoFAD3* (CL1435.Contig 3), were decreased by 5-fold and 10-fold, respectively, from Stage 2 to Stage 6 (Fig. 5B). These *CoFAD* gene expression patterns were consistent with the significant decreases in C18:2 and C18:3 FA levels in the oil tea seeds over different developmental stages (Fig. 5C). Other plastidic desaturases, including FAD6, 7, and 8, and ER-localized FAD2 or FAD3, encoded by *CL5844.Contig2*, *CL18882.Contig3*, *CL9324.Contig2*, and *Unigene10772*, displayed increasing trends throughout seed development, and these genes may be related to the synthesis of membrane phospholipids or galactolipids (Fig. 5D). We also predicted CoFAD3 localization in the ER membrane (Supplementary Data Fig. S17). When we transiently expressed *CoFAD2* in tobacco leaves and examined the lipid profile, increased 18:2 FA contents were observed (Fig. 5E). When *CoFAD3* was transiently expressed in tobacco leaves, we observed greater 18:2 and 18:3 FA contents in the leaves compared with the vector control. These findings suggested that CoFAD2 and 3 are functionally active (Fig. 5E).

**Figure 6:**
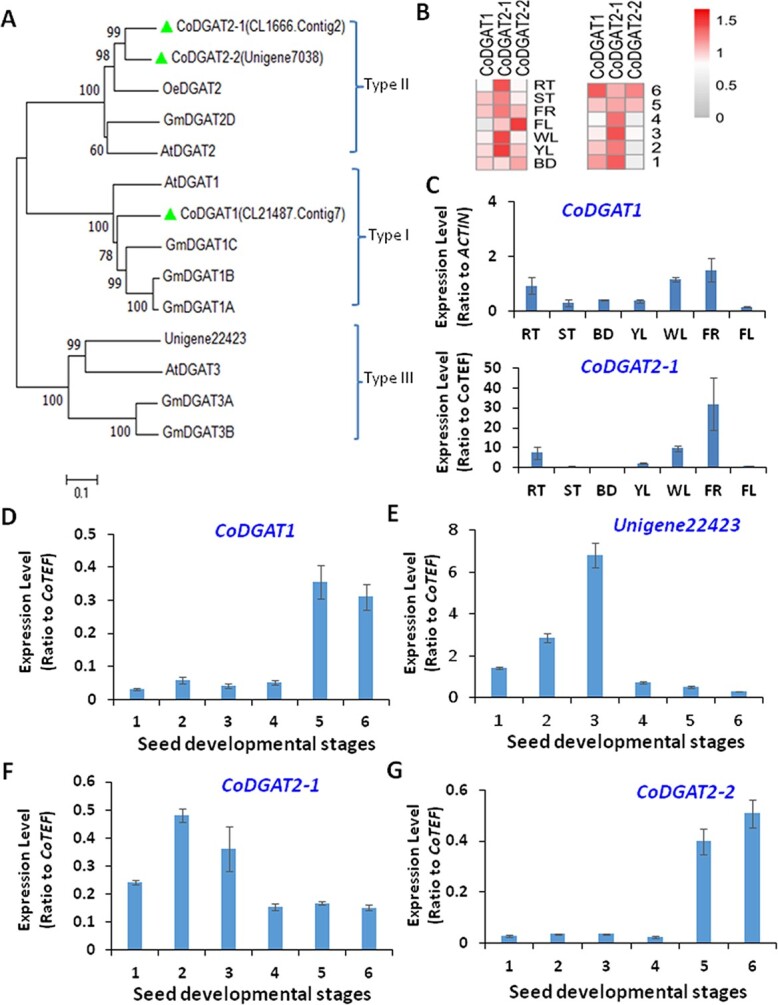
Identification of *CoDGAT* genes from transcriptome of developing oil tea seeds. (A) Phylogenetic analysis of several CoDGATs from transcriptome data on developing oil tea seeds. (B) Heat map analyses of expression profiles of these *CoDGAT*s in seeds at various developmental stages or in different tissues. (C, D) qRT–PCR verification of *CoDGAT1* (C) and *CoDGAT*2 (D) expression in different tissues of oil tea tree. (E) Verification of *CoDGAT*s in oil tea seeds at various developmental stages. Heat maps were generated using transcriptome data and made by using the MeV program. RT, roots; ST, stems; BD, buds; YL, young leaves; WL, old leaves; FR, fruits; FL, flowers.

**Figure 7:**
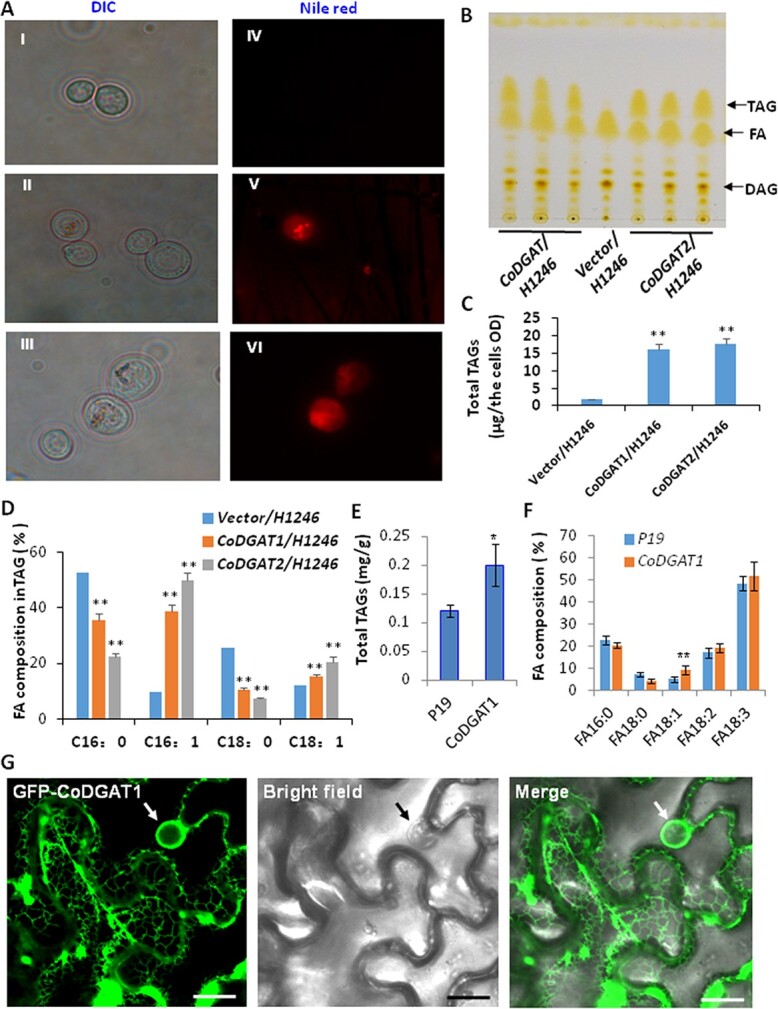
Functional characterization of *CoDGATs* in yeast cells and tobacco plants. The quadruple mutant *Saccharomyces cerevisiae* strain H1246 and the wild-type strain YPH499a were transformed with *CoDGAT* genes and empty pYESDEST52 vector (control). (A) Nile Red staining of oil drops in yeast mutant strain H1246 cells expressing empty pYESDEST52 vector (I, IV), *CoDGAT1* (II, V), or *CoDGAT2* (III, VI). (B) Visualization of DAG, FAs, and TAG of transformed and control strains on TLC silica plate. (C) Total TAG production in yeast mutant strain H1246 cells expressing *CoDGAT2*, *CoDGAT1*, or empty vector. (D) FA compositions of TAG in *CoDGAT1*-, *CoDGAT2*-, and empty vector-yeast mutant strain H1246 cells. (E) Increased TAG contents in tobacco leaves expressing *CoDGAT1* as compared with those expressing empty vector P19. (F) Fatty acid composition in TAGs from tobacco leaves expressing *CoDGAT1* as compared with those expressing empty vector P19. (G) Subcellular localization of GFP-*CoDGAT1* in tobacco epidermal cells. Left, fluorescence image of GFP-CoDGAT1 in the ER; middle, bright-field image; right, merged image. Scale bars = 30 μm. Heat maps were generated using transcriptome data with the MeV program. Data represent three independent experiments and are shown as means ± standard deviation (*n* = 3). Significance differences between nodule and root are shown as ^*^P < .05; ^**^P < .01 (Student’s *t*-test).

### Functional characterization of CoDGATs in triacylglycerol biosynthesis

The deep analysis of the transcriptome of developing oil tea seeds showed four *CoDGAT* genes whose encoded proteins were clustered with soybean, olive, and *Arabidopsis* DGAT1, 2 and 3 (Fig. 6A). *CL21487.Contig7*, *CL1666.Contig2*, *Unigene7038*, and *Unigene22423* were named *CoDGAT1, CoDGAT2-1*, *CoDGAT2-2*, and *CoDGAT3*, respectively (Fig. 6B). The highly expressed *CoDGAT1* and *CoDGAT2-1* (named *CoDGAT2* hereafter) in developing seeds were further functionally characterized. The expression patterns of *CoDGAT1* and *CoDGAT2* at six development stages were examined via qRT–PCR, and the results were in accordance with the RNA-Seq outcomes. Both *CoDGAT* genes exhibited the highest expression at Stages 5 and 6, which is consistent with the stages of the highest oil contents (Fig. 6C). We next characterized the *in vitro* functions of CoDGATs in yeast cells. *CoDGAT1* and *CoDGAT2* were expressed in the TAG-deficient quadruple mutant yeast (*Saccharomyces cerevisiae*) strain H1246 individually, and large oil bodies were observed in *CoDGAT1-* and *CoDGAT2*-expressing H1246 cells, while only minimal oil bodies were found in control H1246 cells expressing empty vector after Nile Red staining (Fig. 7A). Separation of TAGs from the total lipid extracts in these yeast mutant cells by thin-layer chromatography (TLC) confirmed increased TAG levels in both *CoDGAT1*- and *CoDGAT2*-expressing H1246 cells compared with H1246 cells expressing empty vector (Fig. 7B and C). Analysis of the FA composition of TAGs separated by TLC revealed that *CoDGAT1* and *CoDGAT2* expression facilitates the biosynthesis of TAGs with 16:1 and 18:1 fatty acyl chains but decreased TAGs with 16:0 and 18:0 fatty acyl chains, indicating that both CoDGAT1 and CoDGAT2 use 16:1-CoA and 18:1-CoA as acyl donors for TAG synthesis in yeast cells (Fig. 7D)*.* Moreover, the functions of CoDGAT were further analyzed by the tobacco infiltration technique. According to TLC analysis of the infiltrated tobacco leaves, CoDGAT1 increased the TAG amount by 18% compared with control leaves (Fig. 7E). FA analyses showed a significant increase in the 18:1 proportion but a slight decrease in the 18:0 proportion in total TAGs of *CoDGAT1-*overexpressing leaves compared with these of the P19 control (Fig. 7F).

We also examined the subcellular localization of a type I CoDGAT1. GFP-CoDGAT1 fusion driven by a cauliflower mosaic virus 35S promoter was transiently expressed in epidermis of tobacco leaves. Confocal microscopy showed that GFP-CoDGAT1 signals were primarily localized in the ER, and this finding was further supported by chloroplast autofluorescence (Fig. 7G). Due to the high similarity in protein sequences, we posited that both CoDGAT1 and 2 were primarily associated with the ER. Since TAG is synthesized and assembled in the ER, topological analysis of the CoDGAT1 protein sequence and its predicted localization in the ER are consistent with its function as a transmembrane protein (Supplementary Data Fig. S18).

### Regulation of carbon partitioning into oil production by CoWRI1s in oil tea seeds

**Figure 8:**
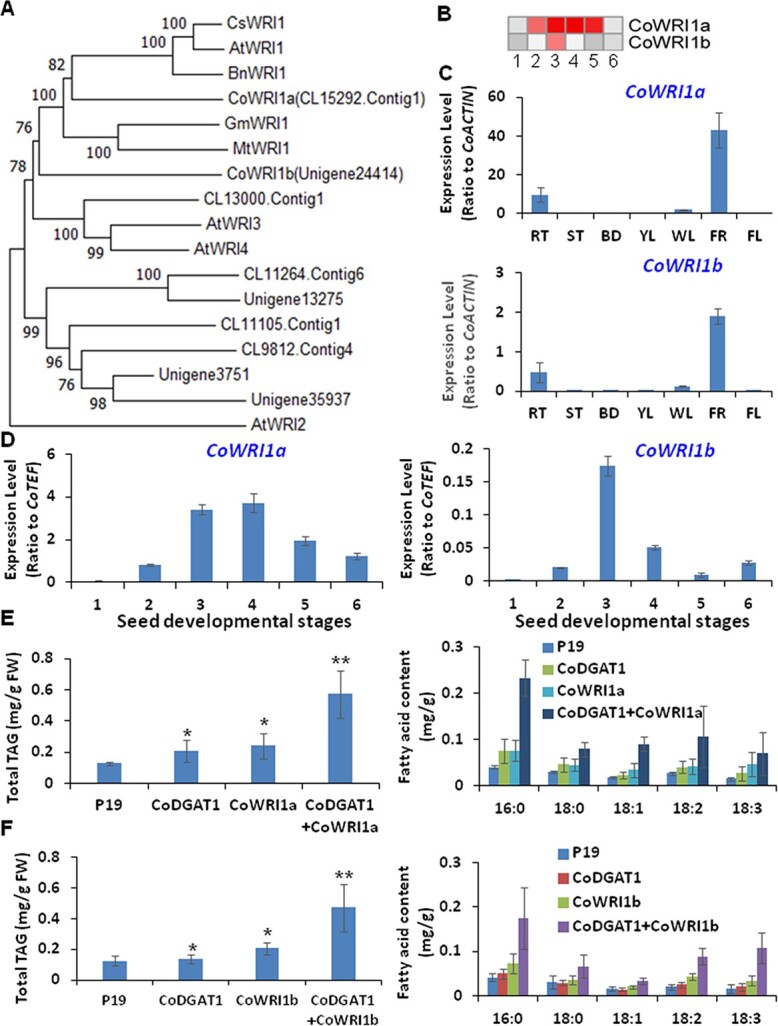
Identification of *CoWRI* genes from the transcriptome of developing oil tea seeds. (A) Phylogenetic analysis of several CoWRIs from transcriptome data on developing oil tea seeds. (B) Heat map analyses of expression profiles of *CoWRI*s in seeds at various developmental stages and different tissues. (C) qRT–PCR verification of *CoWRI1a* and *b* expression in different tissues of oil tea tree. (D) qRT–PCR verification of *CoWRI1a* and *b* in seeds at various developmental stages. (E) Co-expression of *CoWRI1a* with *CoDGAT1* in tobacco leaves with triggered TAG production. Left panel, total TAG; right panel, fatty acid composition of TAG. (F) Co-expression of *CoWRI1b* with *CoDGAT1* in tobacco leaves with triggered TAG production. Left panel, total TAG; right panel, fatty acid composition of TAG. Heat maps were generated using transcriptome data with the MeV program. Data represent three independent experiments and are shown as means ± standard deviation (*n* = 3). Significance differences between nodule and root are shown as ^*^P < .05; ^**^P < .01 (Student’s *t*-test).

Oil biosynthesis in oilseed crops can be regulated at transcription level with regard to source-to-sink carbon partitioning into oils, proteins, or starches as seed storage substances [32, 33]. WRI1 is the most important AP2 transcription factor regulating source carbohydrate glycolysis and carbon partitioning into plastid fatty acid biosynthesis, or even further to TAG production in the ER [32, 33]. Phylogenetic analysis enabled us to identify two AtWRI homolog genes, *CoWRI1a* and *b*, both of which were more specifically expressed in fruit harboring the developing seeds among seven examined tissues and organs, with *CoWRI1a* as the major one as indicated by transcriptome data (Fig. 8A and B). qRT–PCR also verified that both *CoWRI1a* and *CoWRI1b* were highly expressed at Stages 3 and 4 of developing seeds when seed filling started, and actively participated in seed filling (Fig. 8C and D). Their expression patterns are highly consistent with late glycolysis genes (*ALDO*, *G3PDH*, *G3PDH*, *PGK*, *ENO*, and *PK*) and most FA synthesis genes (Fig. 3). To verify the functions of CoWRI1a and b, we further used tobacco leaves overexpressing both *CoWRI1a* or *CoWRI1b* with *CoDGAT1*. Overexpression of *CoWRI1a* or *b* or *CoDGAT1* alone could clearly increase oil production in tobacco leaves (*P* < .05) (Fig. 8E and F). However, when *CoWRI1a* or *b* was co-transformed and co-expressed together with *CoDGAT1* in tobacco leaves, a significant increase in oil production was observed (Fig. 8E and F). The additive or synergistic effects of *CoWRI1a* or *CoWRI1b* with *CoDGAT1* indicated that they are metabolically connected but have different roles in oil production. As in most studies, overexpression of *CoWRI1a* or *CoWRI1b* enabled plants to produce more fatty acids and G3Ps for DGAT to synthesize TAGs.

### Correlation between oil production with *CoSAD/FAD*s, *CoDGAT*s, *CoWRI1*s in 14 oil tea varieties

**Figure 9:**
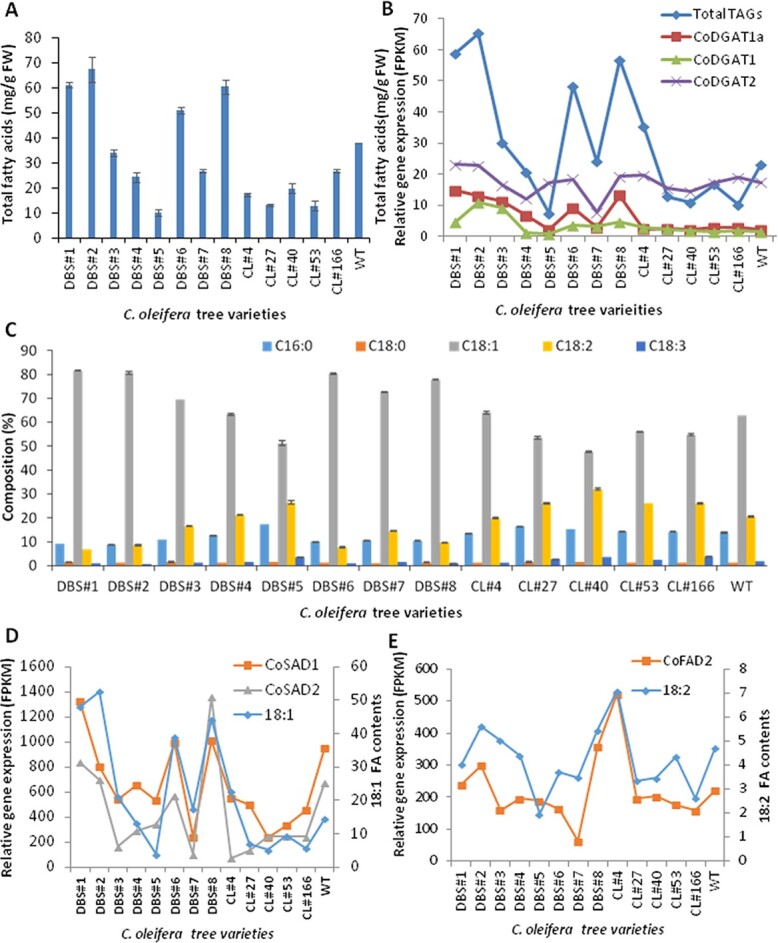
Correlations between oil production and expression patterns of *CoFAD*s, *CoSAD*s, and *CsDGAT*s in seeds of 14 *C. oleifera* varieties. (A) Total TAG contents in seeds of 14 *C. oleifera* varieties. (B) Correlation analysis between total TAG content and expression levels of *CoDGAT*s. (C) Fatty acid compositions in near-mature seeds of 14 *C. oleifera* varieties. (D) Correlation analysis between 18:1 FA content and expression levels of *CoSAD*s. (E) Correlation analysis between 18:2 FA contents in near-mature seeds of 14 *C. oleifera* varieties and their expression levels of *CoFAD*s. Data represent three biological experimental repeats. The gene expression data are based on qRT–PCR results of all tea varieties.

To verify the importance of these *CoSAD*s, *CoFAD*s, and *CoDGAT*s in oil FA composition and production, Stage 5 seeds from 14 local oil tea trees were collected for an association study. These 14 local oil tea varieties were collected at the same time for seed oil and RNA analyses. The seed oil content and FA composition analyses of 14 oil tea varieties showed that the contents varied significantly but were largely consistent with the expression levels of *CoSAD1* and *CoSAD2*, *CoFAD2* and *CoFAD3*, and *CoDGAT1* and *CoDGAT2* in their seeds (Fig. 9). A close positive correlation between the 18:1 contents and expression levels of *CoSAD1* and *2* was observed. In addition, a positive correlation was noted between 18:2 and 18:3 contents and *CoFAD2* and *3* expression levels (Pearson correlation coefficient, *P* > .7) (Fig. 9). Furthermore, the total oil contents and expression levels of *CoDGAT1* and *2* were clearly and positively correlated (Pearson correlation coefficient, *P* > .7) (Fig. 9).

## Discussion

To meet the fast-increasing demand for edible oils from the growing population in China, expanding planting acreage, applying new breeding technology, and investing in basic research on oilseed crops, such as oil tea trees, have started to harvest fruits, such as the recent publication of oil tea tree genome sequences [17, 18]. Still, how to increase oil productivity and meanwhile to improve nutritional quality of edible oils are the major concerns of the Chinese government for national food security. To gain an in-depth understanding and genetic improvement of oil tea trees in terms of oil yield and quality, this study integrated metabolite profiling, transcriptome analysis, and functional characterization to decipher the critical subcellular pathways and key metabolic and regulatory genes. The dissection of expression patterns of genes involved in glycolysis in the cytoplasm, FA biosynthesis in the chloroplast, and TAG synthesis and assembly in the ER of developing oil tea seeds unveiled different features of oil tea seeds, as compared with other well-known oilseed crops, such as oil palm (*Elaeis guineensis*) and olive (*Olea europaea*) trees. Furthermore, we functionally characterized two major plastidic *CoSAD*s, ER *CoFAD2* and *CoFAD3*, and two ER *CoDGAT*s for their coordinative up- or downregulation for cooperation in high 18:1 oil production in oil tea seeds. Two major *CoWRI1*s that could activate glycolysis and fatty acid biosynthesis in developing oil tea seeds were also functionally characterized.

### Dynamic changes of seed storage substances and differential expression of glycolysis genes in developing seeds

Quantification of soluble sugars and starch indicated that soluble starch was maintained at relatively low levels in developing oil tea seeds, as most of the starch was converted into soluble sugars. A considerable number of soluble sugars was partitioned through glycolysis into oil biosynthesis in seeds of oilseed plants [32]. With the increasing contents of soluble proteins in small portions, oil accumulates continuously at seed filling stages and accounts for a significant portion of seed storage substances. While the total oil contents increased markedly throughout oil tea seed development, we observed significant increases in the 18:1 proportion at Stages 2–6 but reduced 18:2 and 18:3 levels in developing oil tea seeds. These findings inspired us to decipher the dynamic changes in these developing oil tea seeds using RNA-Seq analysis.

Glycolysis is the central pathway of carbon metabolism in oilseeds because it converts source sugars into the precursors for TAG and protein biosynthesis at sites where ATP is also produced. The glycolytic pathway is the primary carbon source and produces power for lipid synthesis. In developing oil tea seeds, ALDO and GAPDH were each represented by at least three expressed transcripts, and these transcripts were highly expressed in developing seeds from the young to maturation stages, suggesting the importance of these two genes in oil production, similar to those in oil palm [34]. Increased ALDO levels combined with reduced TPI and GAPDH levels are potentially indicative of changes in carbon flux equilibrium in glycolysis, and amino acid metabolism also seemed to be tightly linked with oil production [34]. Reduction in TPI activity in a yeast knockdown mutant resulted in a 19% increase in lipid content. In contrast, yeast strains overexpressing oil palm ALDO and G3PDH showed 16 and 21%, more lipid content, respectively, compared with GAPDH [25, 35]. Thus, highly expressed *ALDO*, *TPI*, and *G3PDH* genes are also required for oil production in oil tea seeds.

Some plant SUSs, such as *CoSUS1*, are involved in starch degradation for glycolysis and carbon flux into FA and amino acid synthesis pathways, whereas others, such as *CoSUS2*, are involved in sucrose synthesis. Two *SUS* genes, *Unigene41968* (*CoSUS1*) and *CL19004.Contig1* (*CoSUS2*), were highly expressed at the early seed developmental stages, where they displayed opposite patterns. We posited that the sucrose transported to oil tea seeds is mainly hydrolyzed via CoSUS1 rather than INV and channeled into glycolysis as a carbon source for lipid biosynthesis. The expression of an INV (*CL170.Contig4*) and amylase (*CL3151.Contig4*) was continuously increased over the seed developmental stage until maturation, potentially indicating a vital role of these genes in the maintenance of the low level of starch in oil tea seeds, thus ensuring major carbohydrate flux for oil synthesis and storage proteins in the seeds [21–23]. This finding is consistent with the downregulation of *SUS2* in high-oil-palm lines compared with low-oil palms at late developmental stages [36]. The reduced *CoSUS2* expression levels may lead to more lipid accumulation due to carbon channeling away from starch but towards oil biosynthesis. The downstream glycolysis pathway, with only one isoform of each of the *PGK*, *PGM*, *ENO*, and *PK* genes, was highly expressed; they may be also positively regulated by CoWRI1s, since WRI1 is the principal regulator activating late glycolysis and early FA synthesis in oilseed plants [12, 32]. An increased quantity of the carbon flux channeled into FA synthesis via WRI-dependent upregulation of multiple source genes ensured the supply of precursors for increased oil production [12]. Indeed, the expression patterns of *CoWRI1a* and *b* in developing oil tea seeds were consistent with those of late glycolysis genes.

### Active plastidic fatty acid and endoplasmic reticulum triacylglycerol biosynthesis in developing oil tea seeds

The plastidic FA synthesis pathway is a key pathway for oil production in oilseed plants. This pathway is vital for these high-oleic-acid oilseeds, given that oleic acid is mainly synthesized in the plastids before being transported into the ER and incorporated into TAGs. Most genes involved in this FA synthesis pathway were identified and differentially expressed during oil tea seed development. These genes included *CoKAS I*, *CoKAS III*, *CoKAR*, and *ACCase*. ACCase consists of three nuclear-encoded subunits (biotin carboxyl carrier protein, biotin carboxylase, and carboxyl transferase α-subunit, and a plastid-encoded carboxyl transferase β-subunit) and catalyzes the first committed formation of malonyl-CoAs for FA synthesis [11]. Clearly, the transcript levels of multiple *ACCase*s increased during seed development, indicating that these genes are critical for oil production. The oil content is increased 5-fold in plants via overexpression of *ACCase* in the plastid and *ACCase* expression levels are tightly linked with oil production in many oilseed plants [11, 37]. At least one set of highly expressed FA synthetic genes were highly expressed in developing oil tea seeds: *MAT*, *KASIII*, *KASI*, *KASII*, *SAD*, *FatA*, *FatB*, and *SAD* in chloroplasts, and the galactolipid synthesis genes *MGD*s and *DGD*s in chloroplasts. Some of these early FA synthesis genes may be targeted by CoWRI1s [23]. *GPAT*s, *LPAAT*s, *PAP*s, *PDAT*s, *DGAT*s, and *FAD*s as well as oil body protein genes *OLE*s, *CLO*s, and *SEIPIN*s, and TAG lipase genes *DAGL*, *TAGL*, and *MAGL*, in the ER can be regulated by other transcription factors, such as LEC1 and 2 and FUS3 [33]. Surprisingly, at least 10 *OLE* gene transcripts were identified in the transcriptome, and most of them were highly expressed in oil tea seeds.

### Differentially expressed *SAD*s/*FAD*s for high-oleic-acid tea oil production

The hallmark of oil tea seeds with high-quality edible oil is an extremely high content of 18:1, lower levels of saturated FAs (16:0 and 18:0), and ideally high levels of 18:2 and low levels of 18:3. In light of the current study it can be suggested that high plastidic *CoSAD*s activity and reduced transcript levels of *CoFAD2* and *CoFAD3* in the ER during oil tea seed development likely account for the accumulation of a high 18:1 level.

In the plastid, stearoyl-ACP is desaturated to oleoyl-ACP (18:1-ACP) by SADs. Overexpression of *SAD*s from oilseed plants resulted in high levels of 18:1. By contrast, mutation of *SAD*s repressed 18:1 production in total oils from seeds or other organs of the plants [11]. Overexpression of *CoSAD*s in yeast cells also confirmed the functions of these two highly expressed *CoSAD*s in developing oil tea seeds*.* Here, 18:1 is exported from plastids to the cytosol, transported into the ER and incorporated into PA, DAG, and phospholipids. Phospholipids, such as PC and phosphatidylethanolamine (PE), are further desaturated to 18:2- or 18:3- phospholipids by the action of FAD2 and FAD3, which are transmembrane proteins that exert their enzymatic activity on these membrane glycerolipid substrates [11, 38, 39]. Overexpression of *CoFAD3* in tobacco leaves also induced increases in 18:3 accumulation. Mutation of *FAD2* and *FAD3* in *Arabidopsis*, soybean, and rapeseeds resulted in significantly increased oleic acid levels in oilseeds [30, 39, 40]. We demonstrated that several highly expressed *CoSAD*s in plastids enhanced 18:1 biosynthesis, whereas rapidly reduced expression levels of *CoFAD2* and *CoFAD3* genes in the ER suppressed the conversions of 18:1-PC/PEs into 18:2- or 18:3- phospholipids. The low proportion of 18:2 and even lower 18:3 content in tea seed oil could be caused by a reduced ratio of *CoFAD2*/*CoFAD3* transcript levels, which is worthy of further investigation. The analysis of seed oil composition of 14 local oil tea tree varieties and transcript levels of *CoSAD1* and *2* as well as *CoFAD2* and *3* also suggested a close positive relation between 18:1 content and *CoSAD1* and *2* expression levels, as well as between 18:2 and 18:3 contents and *CoFAD2* and *3* expression levels (Fig. 9). These findings are similar to those noted in the olive tree, which produces oil with high oleic acid. In olive trees, *SAD2* is highly expressed in the mesocarp and seed, and the major *FAD2* and *FAD3* genes exhibit low and decreasing expression levels throughout seed development. These characteristics are believed to be responsible for the composition of olive oil [41, 42].

### Triacylglycerol assembly via DGATs and weak acyl editing on triacylglycerols in oil tea seeds

The renewed synthesis and incorporation of new acyl-CoAs into PC occurs via PLA2 and acyl-CoA:lyso-PC acyltransferase (LPCAT), which play important roles in acyl editing on PCs or phospholipids. Generally, the *sn-1* position of PC is esterified with saturated or monounsaturated FA, and *sn-2* is esterified with polyunsaturated FAs (PUFAs). Acyl editing on PC is not only fulfilled through FAD2 and FAD3 but also achieved through deacylation and reacylation modification, which involves PLA2–LPCAT cycles. The PLA2–LPCAT cycle is very active in oilseeds containing high levels of PUFAs in TAGs, given that LPCATs prefer to use 18:2- or 18:3-acyl CoA for PC synthesis [11, 40]. LPCAT transfers the newly synthesized acyl-CoAs from the plastids, mostly 18:1 by highly active SDAs, into the lyso-PCs generated by PLA2 to generate PCs with both oleoyl chains. However, in developing oil tea seeds, both *PLA2* and *LPCAT* transcripts were highly expressed only at the early developmental stages (Stages 1 and 2) but repressed at the late developmental stages, when seed filling occurs.

TAGs assembled in the ER are mainly formed via two mechanisms: using fatty acyl-CoAs as acyl donors and DAGs as acyl acceptors via the functions of DGATs or using PCs as acyl donors and DAGs as acceptors via the functions of PDATs [11, 19, 43–46]. Several highly expressed *DGAT*s in developing oil tea seeds, including *CoDGAT1* and *CoDGAT2*, may trigger increased 18:1 production. Both types of CoDGAT may prefer oleoyl CoA over other fatty acyl-CoAs as a substrate for TAG synthesis, which needs to be further verified. The expression levels of *CoDGAT1* and *2* in seeds of 14 local oil tea varieties were clearly correlated with their total oil contents (Fig. 9).

Extensive acyl editing on plant DAG and phospholipids via phosphatidylcholine diacylglycerol cholinephosphotransferase (PDCT) also enable the incorporation of more polyunsaturated FAs into TAGs through PDAT [11]. CDP-choline:DAG cholinephosphotransferase (DAG-CPT) transfers choline from CDP-choline to DAG to generate new PC to recycle DAG into PC; DAG-CPT-PDCT-PDAT forms an important PC-DAG exchange/conversion cycle to enforce the acyl editing on TAGs [11, 45, 47]. The reduced CoPDCT activity could be another reason for the increased 18:1 proportion in TAG in addition to low *CoFAD2/CoFAD3* expression at late seed developmental stages (Supplementary Data Fig. S19). In developing oil tea seeds, *CoPDCT* was downregulated in general; thus, its activity of converting DAG back to the PC pool was reduced, thereby reducing the chance of desaturation of 18:1 on PC (Supplementary Data Fig. S19). The reduced number of *PDCT*, *DAG-CPT*, and *PDAT* transcripts at low expression levels over oil tea seed development indicated that PDCT-mediated acyl editing of TAG may play a minor role in 18:1 accumulation (Supplementary Data Fig. S19). The *Arabidopsis PDCT* mutant contains higher levels of oleic acid but reduced 18:2 and 18:3 levels [12, 47]. Furthermore, compared with highly expressed *CoDGAT*s, *CoPDAT*s that encode the enzymes utilizing PC and DAG as substrates to create TAG were expressed at much lower levels and may contribute less to TAG production in oil tea seeds. By contrast, rapeseed, soybean, and flax seeds contain higher levels of PUFAs, such as 18:2 and 18:3; PDATs are regarded as major contributors to the biosynthesis of TAGs [11, 45, 48, 49]. In general, the higher expression of DGATs and the lower expression of PDATs are likely responsible for the TAGs containing monounsaturated fatty acids [11, 43, 45]. Thus, the lower ratio of *PDAT*/*DGAT* gene expression in developing oil tea seeds may also determine the increased 18:1 percentage in TAGs. Ectopic expression of two *CoWRI1*s, individually or together with *CoDGAT*s, which are assumed not to be directly regulated by WRI1, further confirms that *CoDGAT*s are metabolically linked with CoWRI1-activated genes/enzymes for precursors.

### Comparison of mechanisms underlying high-oleate-oil biosynthesis in oilseeds

The genome sequence of high-oleate oil-producing olive revealed that the duplication and differential expression of *OeFAD2* and *OeSAD* are responsible for the differential accumulation of 18:1 and 18:2 FA in olive seeds [50]. Several *OeFAD2* genes in olive seeds are suppressed by an siRNA, resulting in reduced conversion of 18:1 into 18:2. Meanwhile, neofunctionalization of several *OeSAD* genes led to increased desaturation of 18:0 [50]. The decreased *OeFAD2* and increased *OeSAD* expression explained the accumulation of high levels of 18:1 in olive oil [50]. This situation is quite similar to high-oleate production in oil tea seeds. The genomic bases of different expression patterns of *CoSAD* and *CoFAD* in oil tea seeds are still not clear, and they are not reported as regulation targets of WRIs [11, 12]. However, the exceptional numbers of transcripts encoding oil body proteins, including 45 transcripts for OLEs, 11 for CLOs, 4 for SLOs, and 7 for seipins from individually assembled full-length transcriptome data (Supplementary Dataset S3), may indicate the multiple genetic loci for these oil body genes. Both our transcriptome and genome sequences of oil tea plants showed that there are more transcripts for oil body proteins in oil tea plants than in other oilseed plants, such as 17 *OLE* genes in diploid *Arabidopsis* and 13 in allopolyploid soybean (Supplementary Dataset S4) [18, 51]. OLEs play an essential role in stabilization of the coat surface of oil bodies, and are positively associated with the high oil production in oilseeds, including in tea oil production [18, 51]. Palm oils from mesocarps and kernels are compositionally different. Genome sequences revealed that palm oil biosynthesis is mainly controlled at transcriptional level [52, 53]. The expression of *FatB*, *FAD7/8*, *SAD*, *FatA*, *LACS*, *KASI*, *KASII*, and *KASIII* genes in the chloroplast is critical for higher oil production in oil palm [52, 53]. These genes are tightly regulated by a WRI, which is characterized as a key marker for high-oil-yield oil palm varieties, rather than in date palm [20, 52, 53]. Similar to oil palm WRI, CoWRI1a and b also play key roles in regulating oil biosynthesis in oil tea seeds. Sesame (*Sesamum indicum*) seeds contain a high level of oil, which constitutes ~59% of the dry seed and consists of more unsaturated FA. In sesame seeds *SiKASI* and *SiDGAT2* are two key genes responsible for the saturated/unsaturated fat ratio, whereas *SiFAD2* and *SiSAD* contribute to the natural genetic variation in 18:1 FA [54]. All studies indicate that the expression levels of *SAD*, *FAD*, and *DGAT*/*PDAT* genes are critical for high oil production in oil seed crops and also critical for their oil composition [11, 12, 54]. Our study demonstrated that oil tea plant WRI1s may activate the late glycolysis genes, such as *PK*, and early FA biosynthesis genes, such as *KAS*, *BCCP*, *ACP*, and *ACBP*, in the chloroplast for oil production in oil tea seeds, which is worthy of further investigation.

### Conclusions

Given that the breeding of *C. oleifera* to increase the production of tea seed oils could be seriously limited by poor understanding of the molecular and genetic basis for seed development and oil biosynthesis and regulation, we combined transcriptome and metabolic analyses of developing seeds to decipher the glycolysis and lipid synthetic pathways and genes. Furthermore, we characterized the key FA desaturase genes—*CoSAD*s and *CoFAD*s—that are responsible for the high 18:1, appropriate 18:2, and low 16:0, 18:0, and 18:3 levels in oil seed seeds. Two major CoDGATs that may prefer to synthesize TAGs with oleoyl acyl chains were characterized. Two key oil biosynthesis regulators CoWRI1s are also found in abundance in developing seed, and CoWRI1a and b activate late glycolysis and FA synthesis to direct the source carbon flux towards oil production. These genes are responsible for high-oleic-acid-oil production in oil tea seeds. These key metabolic and regulatory genes not only provide potential molecular tools for metabolic engineering of high-quality edible oil production in oilseed crops or other engineered microorganisms, but also can be developed into useful markers for molecular-assisted breeding of new oil tea varieties with high oil yields and desirable FA compositions.

## Materials and methods

### Plant materials

Developing fruits of *Camellia oleifera* Abel. cultivar ‘Changlin #4’ were used in this study. Twelve-year-old trees grown in Dechang Camellia tree farmer lands, Anhui Province, China, were used for sampling developing seeds of various stages. After the oil tea trees bloomed, seeds at different developmental stages were sampled regularly, and samples representing early embryo, growing embryo, early and late seed filing, and maturation stages were selected for FA, oil, RNA-Seq, and gene expression analysis. Six seed developmental stages, which were counted in weeks after flowering, were about 14, 18, 22, 27, 30, and 36 weeks after flowering, corresponding to the first weekends of May, June, July, the middle of August, early September, and late October in 2016, 2017, and 2018, respectively. For convenience, they were named Stage 1, 2, 3, 4, 5, and 6, respectively. The fruits at 36 week after pollination (WAP) of oil tea cultivar ‘Changlin #4’ were almost completely mature. For each time point, at least 50 young oil tea fruits or 20 older oil tea fruits were collected for the experiments. The oil tea fruits from each tree of the same cultivar in similar sizes from different bunches were collected and randomized to obtain repeat samples for metabolite and RNA analyses. The oil tea fruits and peeled seeds were stored at −80°C for further studies. For the association study, the fruits of 14 other oil tea trees grown in the same garden were also picked (at about Stage 5) in September for the seed oil and RNA analyses.

### RNA extraction, library construction, sequencing, and annotation

For the RNA-Seq analysis, we collected six developmental stages of *C. oleifera* seeds. The six cDNA libraries were sequenced using Illumina RNA-Seq technology, as described previously. Total RNA was extracted using an RNA Extraction Kit (BioTeke, Beijing, China). The Ultra™ RNA Library Prep Kit for Illumina (NEB) was used to synthesize double-stranded cDNAs. Further, cDNAs were prepared for Illumina HiSeq2500 paired-end sequencing. *De novo* assembly and functional analyses of transcripts were done as previously described [15, 16]. For differentially expressed gene (DEG) analysis the assembled contigs with more than 10 reads mapped were chosen. For the expression analysis, the number of clean reads for each contig was calculated and then normalized to reads per kilobase per million reads (RPKM). The expression difference of each contig between different treatments was calculated based on the MARS model using the DEGseq package. Transcriptome annotation was performed using unigenes as query sequence against several databases, including NR, Swiss-Prot, and the Gene Ontology (GO) database. Unigenes were categorized into three broad categories: biological processes; cellular components; and molecular functions. Unigenes were also used to query the KEGG and Pfam databases for specific function identification.

### Gene cloning and vector construction

The open reading frames (ORFs) for *CoDGAT1*, *CoDGAT2*, *CoSAD1*, *CoSAD2*, *CoFAD2*, *CoFAD3*, *CoWRI1a*, and *CoWRI1b* were cloned by PCR. For PCR amplification, gene-specific primers were designed based on the RNA-Seq information of six developmental stages of oil tea seeds. Five micrograms of total RNA was used to synthesize the first-strand cDNA through the first-strand synthesis system Superscript III (Invitrogen, USA) for cloning *CoSAD*s, *CoFAD*s, *CoWRI1*s, and *CoDGAT*s. The ORFs were cloned under PCR amplification conditions with pairs of gene-specific primers. The PCR products were cloned into the Gateway entry vector pDONR221 using BP Clonase (Invitrogen, USA). The resulting pDONR221 constructs were sequenced, and cloned into the destination vector using Gateway LR Clonase (Invitrogen, USA). All constructs were confirmed by sequencing and for transforming yeast strains or plants.

### Yeast expression of CoDGAT proteins and functional assays

For the functional expression of *CoDGAT1* or *2* and *CoSAD1* and *2* in yeast, the quadruple mutant *S. cerevisiae* strain H1246 (W303; MATα *are1-Δ::HIS3 are2-Δ::LEU2 dga1-Δ::KanMX4 lro1-Δ::TRP1 ADE2 ura3*) was used as heterologous host. Stationary H126 cells were transformed using plasmid DNA pYESDEST52-*CoDGAT*s and pYESDEST52-*CoSAD*s through the PEG/lithium acetate method. Yeast cells harboring the empty pYESDEST52 vector were used as negative control. Transformants were carefully chosen on YNB medium lacking uracil and functional characterization of *CoDGAT*s or *CoSAD*s was carried out as described in Unver *et al*. [50]. The intracellular lipid bodies were stained using the Nile Red staining technique and visualized through fluorescence microscopy. Images of the stained cells were captured under a Nikon Eclipse 80I microscope using the emission wavelength of 538 nm and excitation wavelength of 485 nm. Alternatively, total lipids were extracted and separated by TLC and quantification was carried out by gas chromatography (GC).

### Quantification of triacylglycerol and fatty acid composition

Extraction of all kind of lipids and seed TAG amount and composition were determined with little modification according to the methods described in previous studies [32, 55]. Briefly, the three biological replicates of 30 mg oil tea seeds were ground in liquid nitrogen for total lipid extraction. The TAG from yeast cells and tobacco leaves was determined by TLC on a silica plate (SIL GF254, 0.25 mm). The plate was developed with hexane/diethyl ether/acetic acid (80:20:1, v/v/v), as described previously [56]. The amount of TAG and its composition in oil tea seeds was measured as explained in Chen *et al*. [55]. FAs in TAGs from seeds, hairy roots, and yeast cells were analyzed using a GC (Agilent 7890A) system having a flame ionization detector (FID). Triheptadecanoin in toluene (Nu-Chek Prep, Elysian, USA) was used as an internal standard during extraction, the transesterification reaction, and GC analysis. The oil content was calculated relative to 17:0 methyl ester (Sigma–Aldrich, CA, USA).

### Analyses of seed sugar, starch and protein contents

The sugar and starch contents were analyzed via the anthrone assay as described previously [32, 56]. Total seed protein was extracted following the method described previously [32].

### Quantitative RT–PCR analysis of gene expression

Total RNA from six developmental stages of oil tea seeds was extracted and used to prepare first-strand cDNAs. All cDNA samples were further 20-fold diluted with sterilized water for quantitative real-time PCR (qRT–PCR). Gene-specific primers provided in Supplementary Data Table S1 were used for qRT–PCR in 96-well plates (iQ5 Real Time PCR System; Bio-Rad). The *CoACTIN* and *CoETF* genes were used as internal controls. All analyses were performed in three biological replicates with three technical replications.

### Subcellular localization of CoFAD and CoDGATs

GFP-CoDGAT1 and GFP-CoSAD1 fusions were generated by using a Gateway recombination system into the pK7WGF2 vector in combination with GFP at the N-terminus of the genes [55]. Subcellular localization of these GFP fusions was achieved by using the tobacco (*Nicotiana benthamiana*) leaf infiltration system previously reported [55]. Images of GFP-CoDGAT1- or -CoSAD1 fusion proteins were captured with an Olympus confocal microscope using a 63× water-immersion objective with an excitation wavelength of 488 nm and emissions collected at 500 nm.

### Transient expression of *CoDGAT*s, *CoFADs*, and *CoWRI1*s in tobacco plants

Transient expression in tobacco leaves was carried out with some modifications as described previously [56]. *Agrobacterium tumefaciens* cultures harboring the gene coding for the P19 viral suppressor protein and the genes of interest were mixed together. The final concentration of both cultures was 0.125 at an OD of 600 prior to infiltration. For lipid analysis, 20 leaves from five plants were infiltrated with each of the genes individually or in different combinations. Samples for comparison were randomly located on the same leaf. After 5 days of infiltration, tobacco leaf disks (three from each plant) were harvested and stored at −80°C. The TAG from tobacco leaves transiently expressing *CoDGAT1*, or *CoFAD2* and *3*, and *CoWRI1*, either individually or in different combinations, was separated by TLC and analyzed by GC.

### Phylogenetic analysis

Phylogenetic analyses were carried out using the MEGA 7.0 program (http://www.megasoftware.net). The neighbor-joining method was used to construct the phylogenetic tree with 1000 bootstrap trials by MEGA 7.0. The GenBank accession numbers for oil tea genes are: CoSAD1 (MT038368); CoSAD2 (MT038369); CoFAD2a (MT038370); CoFAD3a (MT038371); CoFAD6 (MT038372); delta-24-sterol desaturase (MT038373); CoFAD3b (MT038374); CoFAD2b (MT038375); CoSAD3 (MT038376) CoSAD4 (MT038377); CoDGAT1 (MN810335); CoDGAT2-1 (MN810336); CoDGAT2-2 (MN810337); CoDGAT3 (MN810338); CoPDAT1 (MN810339); CoPDAT2 (MN810340); CoWRI1a (MN810333); and CoWRI1b(MN810334).

### Statistical analysis

All tests were conducted in triplicate. Statistical analysis was performed using Student’s t-*t*est, and *P* values <.05 were considered statistically significant.

## Acknowledgements

We thank other laboratory members for their assistance in the experiments and data analysis. We also thank Mr Zhan from the Dechang oil tea (*Camellia oleifera*) Plant Garden in Luan for providing oil tea germplasm for sampling and preparation. This work was supported by the Key Research and Development Program of Anhui Province (18030701155), the National Key Research and Development Program of China (2018YFD1000601), Anhui Agricultural University, and the State Key Laboratory of Tea Plant Biology and Utilization.

## Author contributions

J.Z. planned and designed the research. J.H.Y, G.B.S, P.L., B.B.C., S.M., C.L., and S.C.Z. performed the experiments and analyzed the data. J.Z. and J.H.Y wrote and edited the manuscript.

## Data and material availability

All data supporting the findings are available and provided in the supplementary data; the material may be available for distribution upon request.

## Conflict of interest

The authors declare that they have no competing interests.

## Supplementary data

Supplementary data is available at *Horticulture Research* online.

## Declarations: Ethics approval and consent to participate

No investigations were undertaken using humans/human samples in this study. No experimental animals were used to conduct any of the experiments reported in this manuscript, and our study did not involve endangered or protected species. Specific permits were not required for the studies. J.Z. should be contacted for future permissions.

## Supplementary Material

Web_Material_uhac087
