# Supplementary material for: Critical metabolic pathways and SAD/FADs, WRI1s, and DGATs cooperate for high-oleic acid oil production in developing oil tea (Camellia oleifera) seeds
Source: Hortic Res. 2022 Apr 21;9:uhac087. doi: 10.1093/hr/uhac087 (PMC9178347; doi:10.1093/hr/uhac087)
Supplement: Web_Material_uhac087 [file web_material_uhac087.zip › CoSAD-FAD-DGAT Supplemental figures JZhao R3.pdf]

Supplemental Figures for:

Critical metabolic pathways and SADs, FADs and DGATs cooperate for high-oleic acid oil production in developing oil tea seeds (*Camellia oleifera*)

Jihong Yang, Beibei Chen, Sehrish Manan, Penghui Li, Chun Liu, Guangbiao She, Shancen Zhao, Jian Zhao*

Supplemental Figure S1. *De novo* assembly and analysis of transcriptome of oil tea developing seeds

a. Venn diagram of assembled transcripts of oil tea seeds with major databases

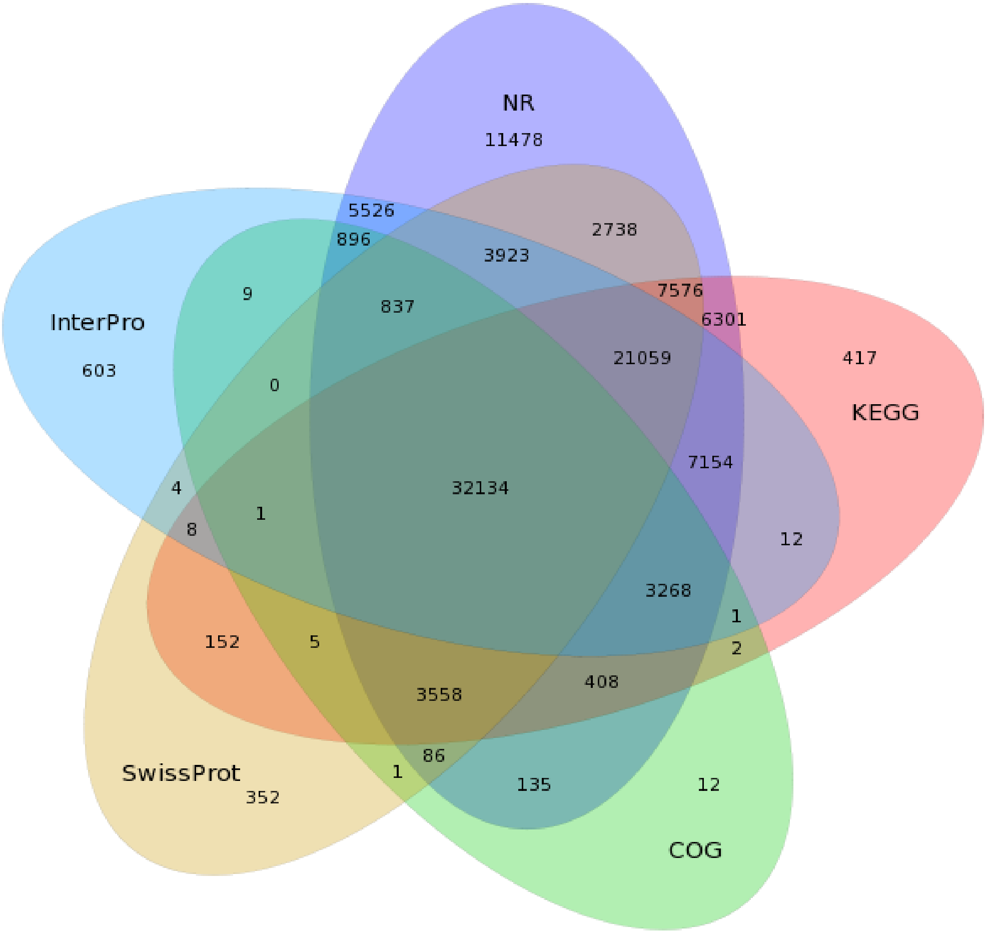

b. Comparison of transcripts of oil tea plant seeds with other plant species

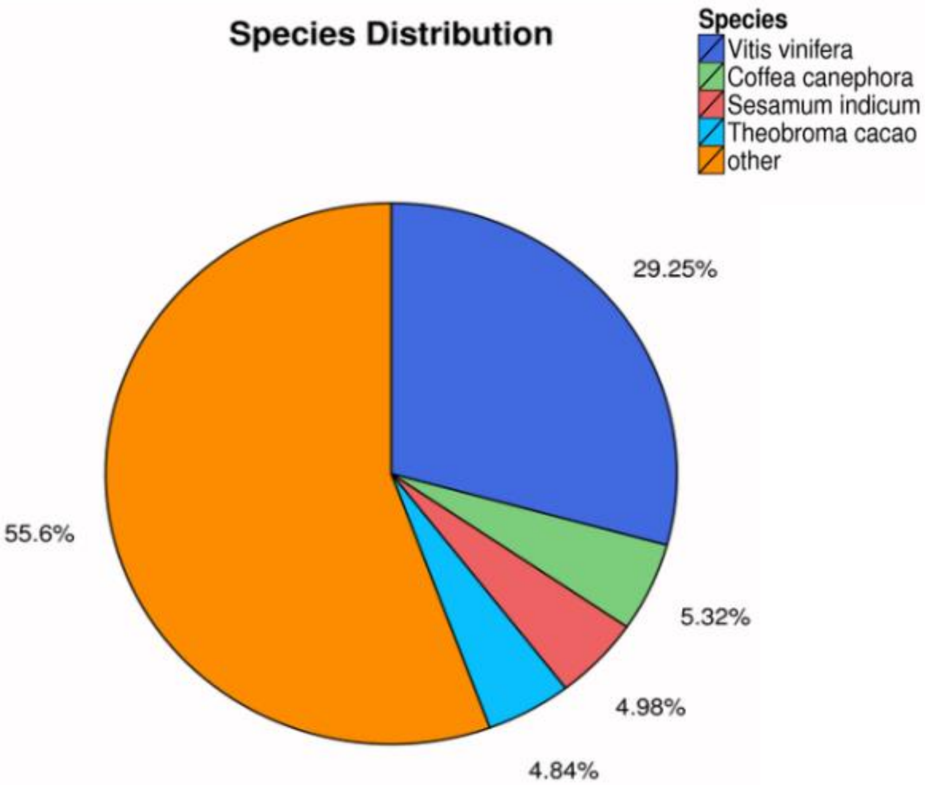

ACCase

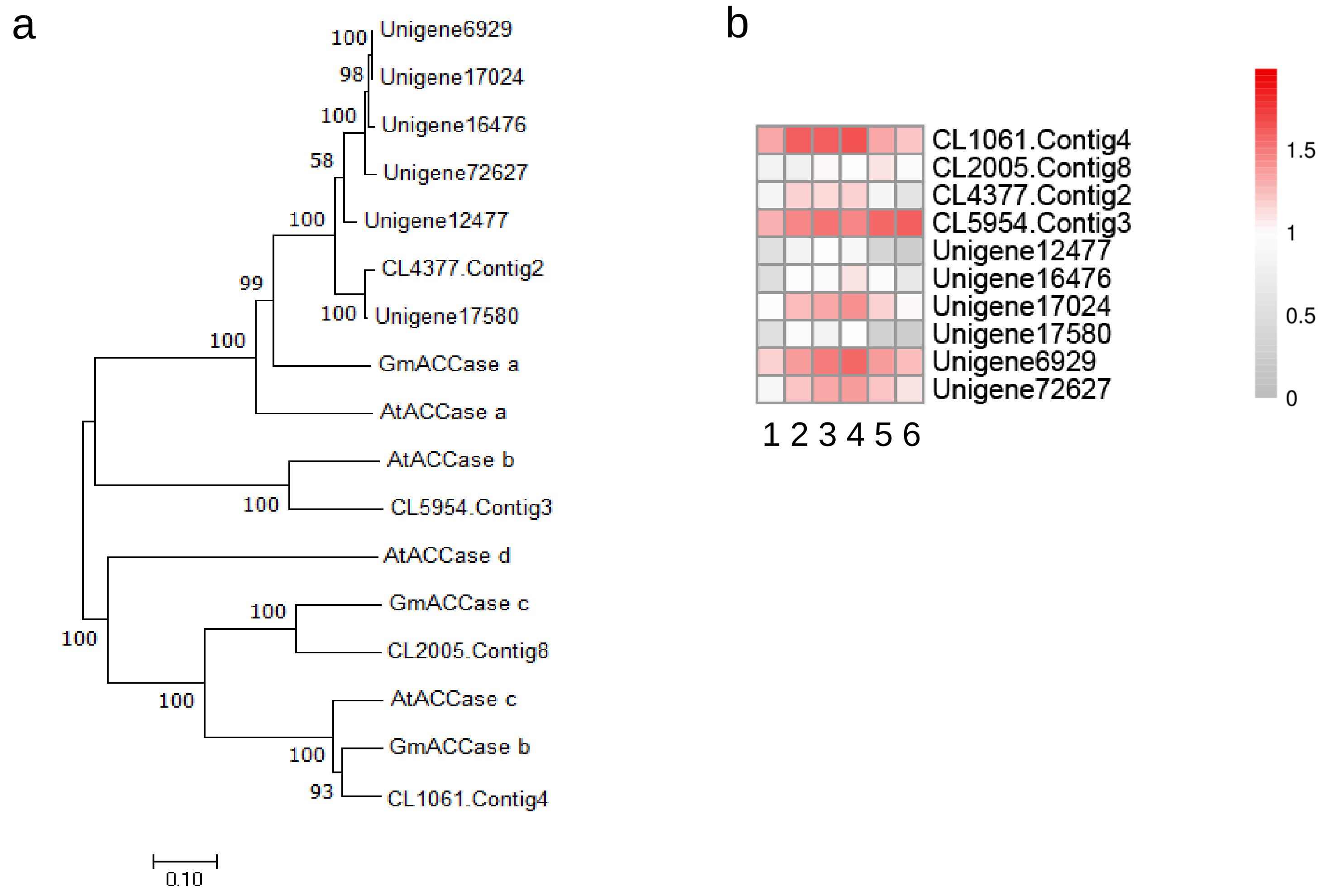

Supplemental Figure S2. Identification and analysis of ACCases from oil tea oil tea developing seeds
(a) Unrooted phylogenetic tree of oil tea (*Camellia oleifera*) for acetyl CoA carboxylases (ACCase) with functionally characterized ACCases from other plants. The alignment was generated using ClustalW and the unrooted phylogram was constructed by the neighbor-joining method in MEGA7 software.
(b) Heatmap analysis of gene expression patterns of ACCase transcripts in oil tea seeds at various developmental stages. Data are from transcriptome of developing seeds, R-Studio was used form making the heatmap.

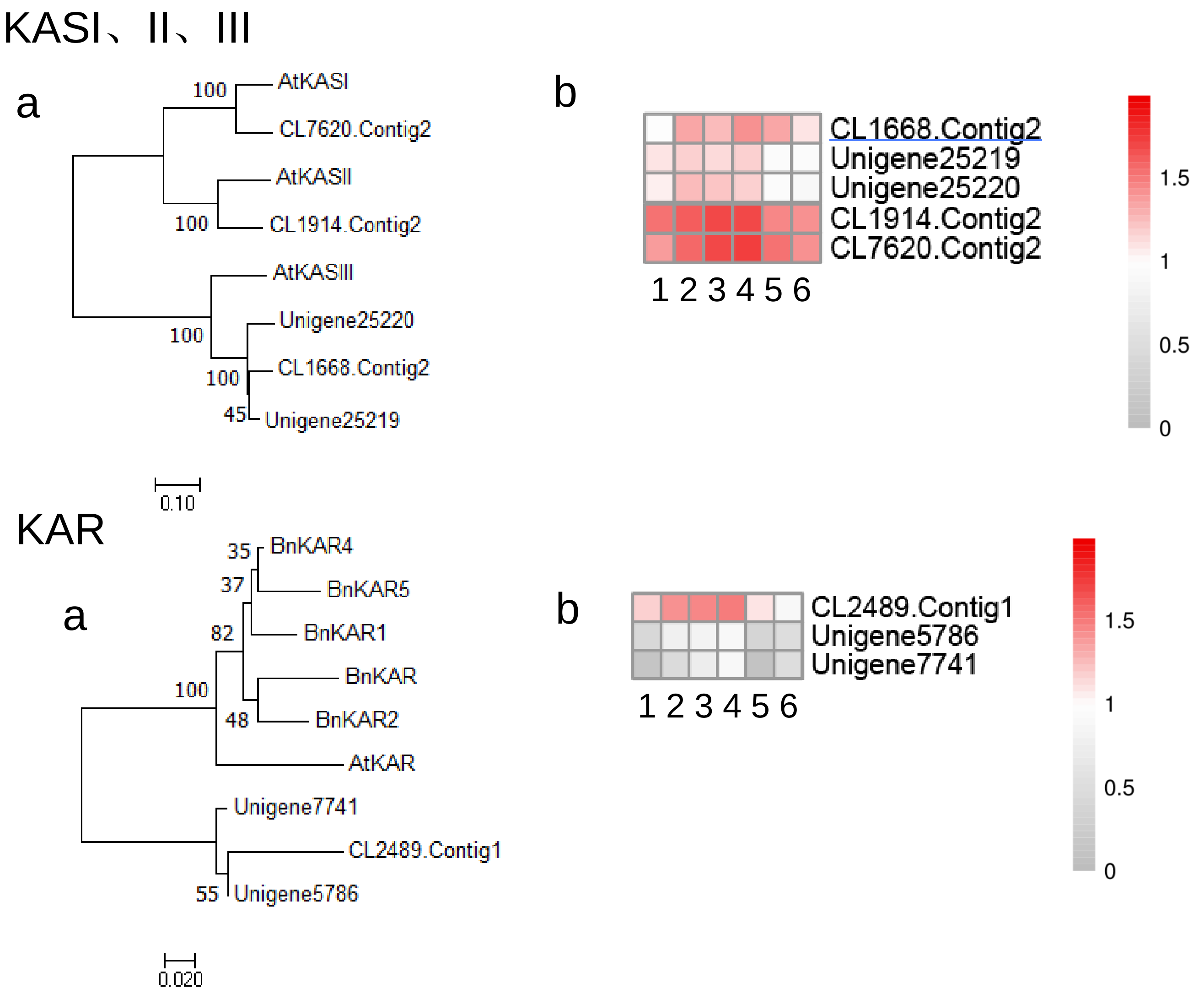

Supplemental Figure S3. Identification and analysis of KASIs, Iis, IIIs, and KARs from oil tea developing seeds
(**a**) Unrooted phylogenetic tree of oil tea (*Camellia oleifera*) for β-Ketoacyl-acyl Carrier Protein Synthase I (KASI), KASII, KASIII, and ketoacyl reductase (KAR) with functionally characterized ones from other plants. The alignment was generated using ClustalW and the unrooted phylogram was constructed by the neighbor-joining method in MEGA7 software.
(**b**) Heatmap analysis of gene expression patterns of KASI, II, III, and KAR transcripts in oil tea seeds at various developmental stages. Data are from transcriptome of developing seeds, RStudio was used form making the heatmap.

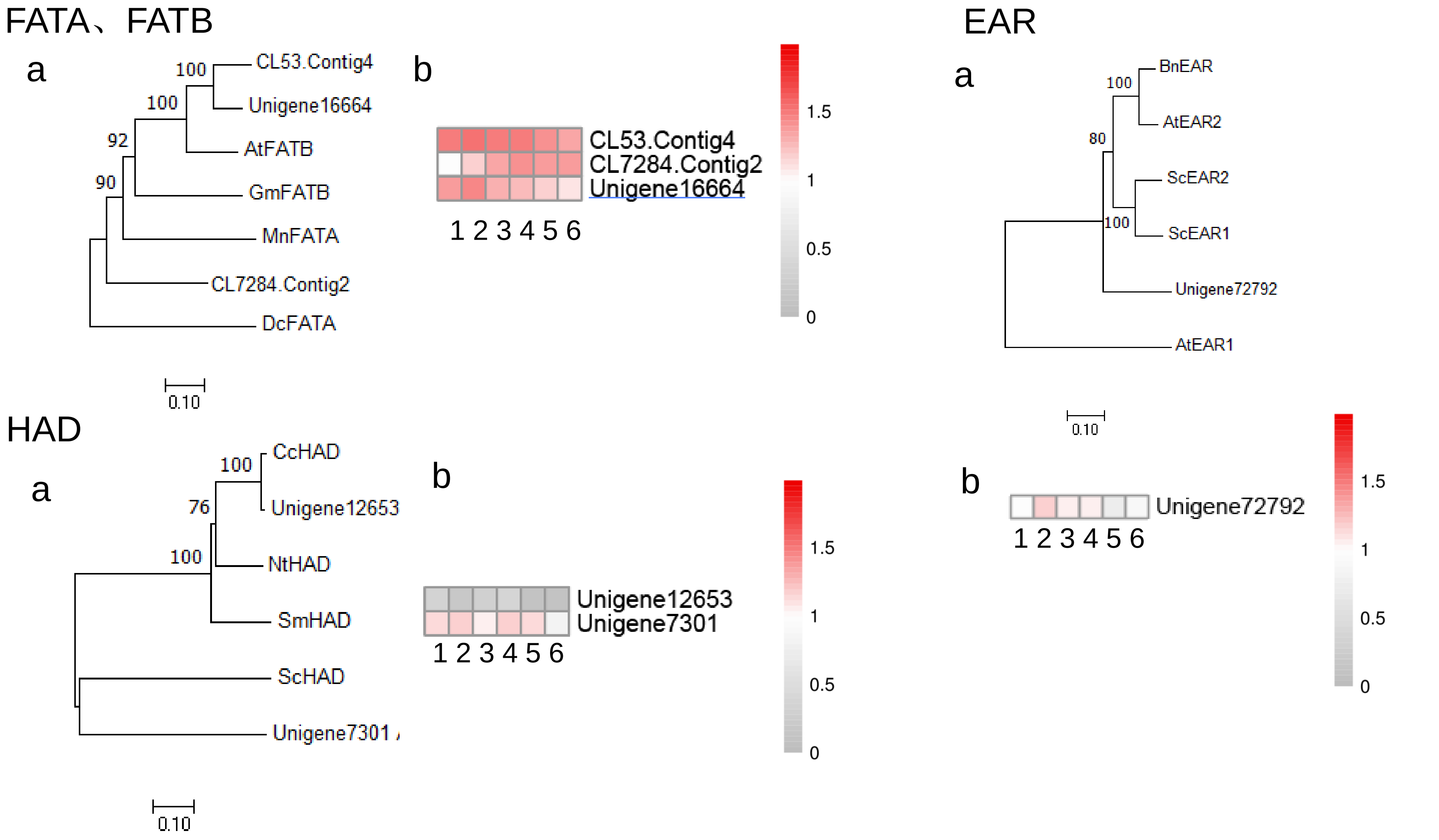

Supplemental Figure S4. Identification and analysis of FATA, FATB , HAD and EAR from oil tea developing seeds

(a) Unrooted phylogenetic tree of oil tea (*Camellia oleifera*) for fatty acyl-acyl carrier protein thioesterase A (FATA) , FATB, and 3-Hydroxyacyl ACP dehydrase (HAD), and enoyl-ACP reductase (EAR) with functionally characterized ones from other plants. The alignment was generated using ClustalW and the unrooted phylogram was constructed by the neighbor-joining method in MEGA7 software.

(b) Heatmap analysis of gene expression patterns of FATA, FATB, HAD and EAR transcripts in oil tea seeds at various developmental stages. Data are from transcriptome of developing seeds, RStudio was used form making the heatmap.

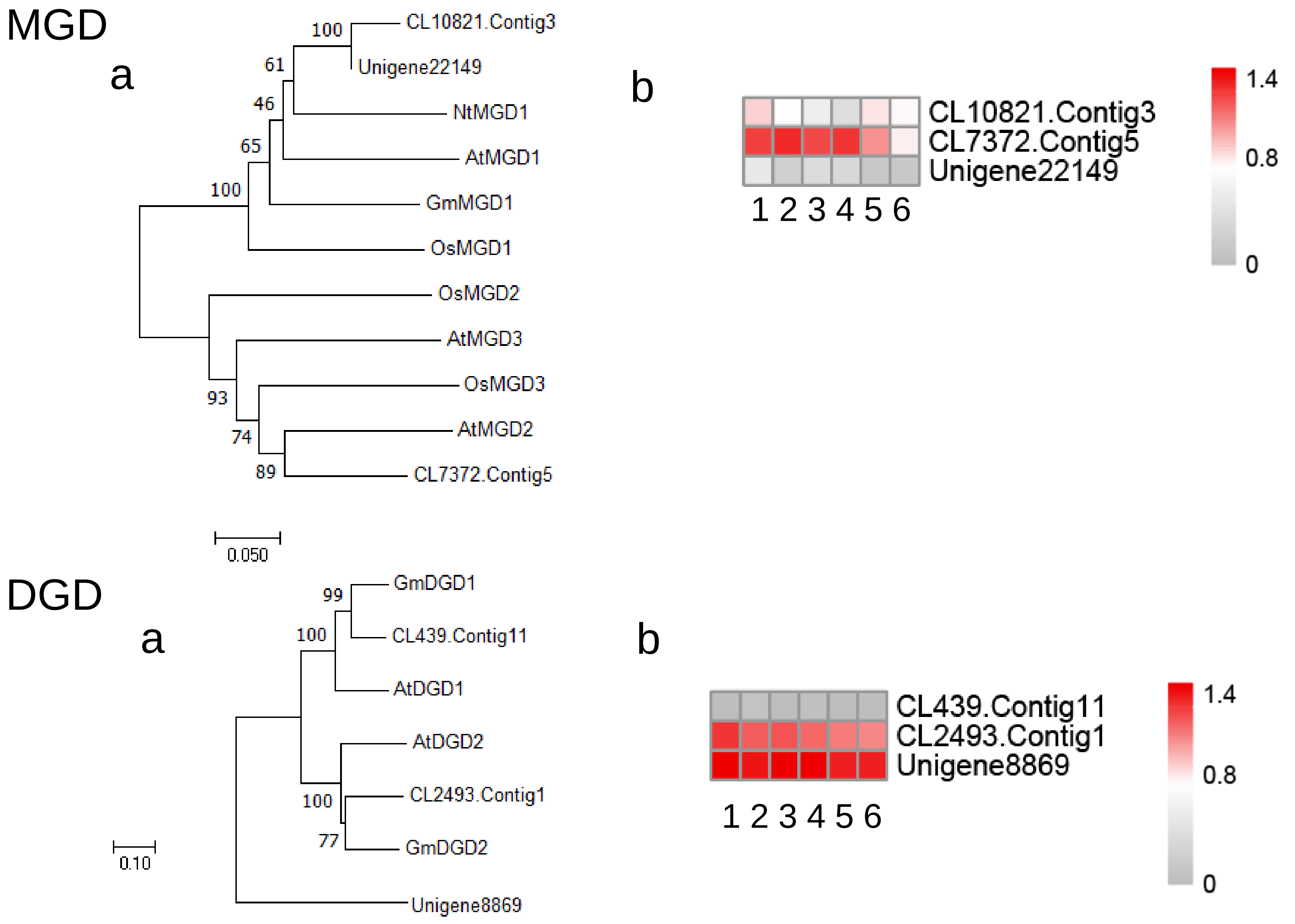

Supplemental Figure S5. Identification and analysis of MGDs and DGDs from oil tea developing seeds
(**a**) Unrooted phylogenetic tree of oil tea (*Camellia oleifera*) for monogalactosyldiacylglycerol synthase (MGD) and digalactosyldiacylglycerol synthase (DGD) with functionally characterized ones from other plants. The alignment was generated using ClustalW and the unrooted phylogram was constructed by the neighbor-joining method in MEGA7 software.
(**b**) Heatmap analysis of gene expression patterns of MGD and DGD transcripts in oil tea seeds at various developmental stages. Data are from transcriptome of developing seeds, RStudio was used form making the heatmap.

TGD

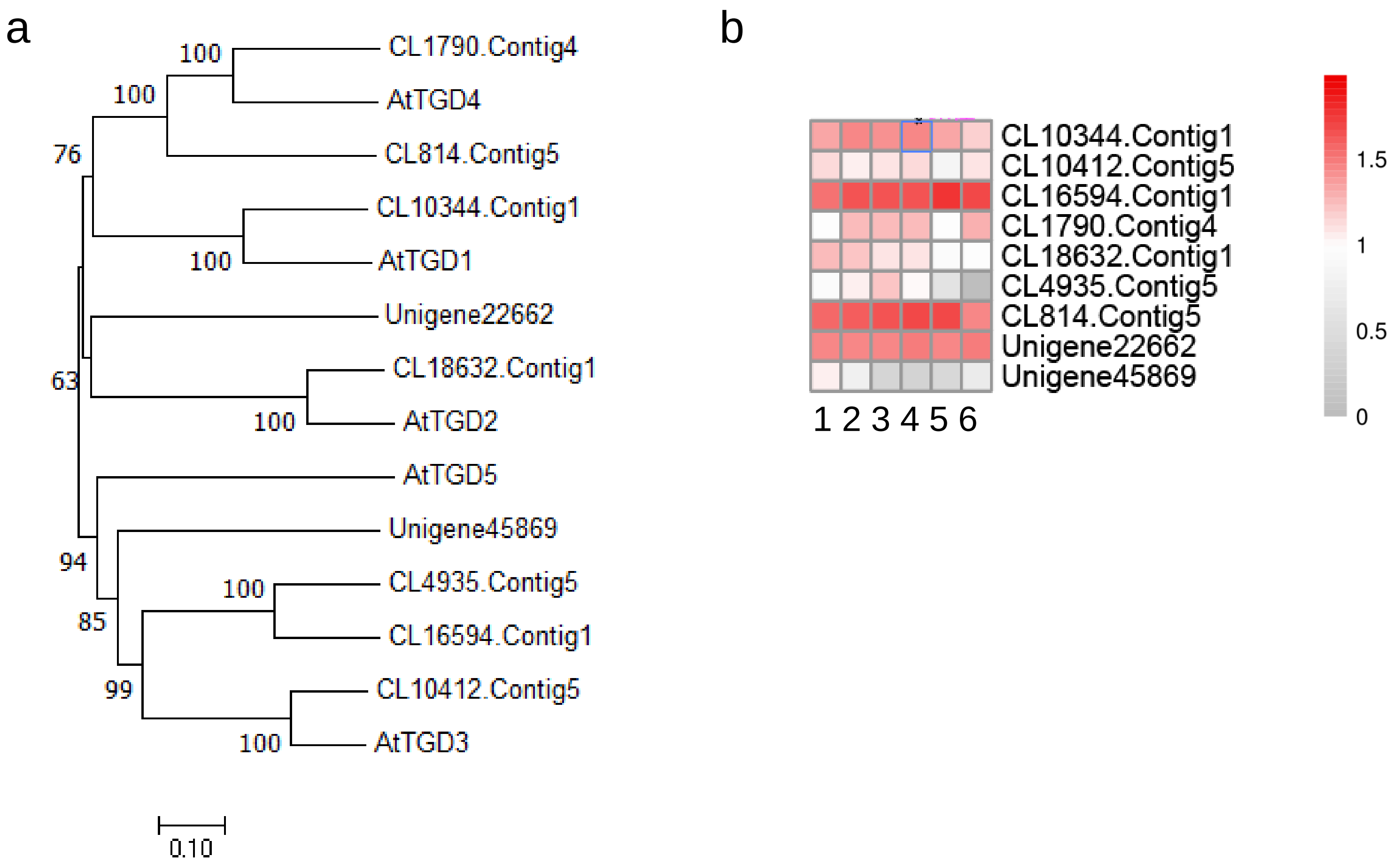

Supplemental Figure S6. Identification and analysis of TGDs from oil tea developing seeds
(**a**) Unrooted phylogenetic tree of oil tea (*Camellia oleifera*) for trigalactosyl diacylglycerol (TGD) with functionally characterized ones from other plants. The alignment was generated using ClustalW and the unrooted phylogram was constructed by the neighbor-joining method in MEGA7 software.
(**b**) Heatmap analysis of gene expression patterns of TGD transcripts in oil tea seeds at various developmental stages.
Data are from transcriptome of developing seeds, RStudio was used form making the heatmap.

GPAT

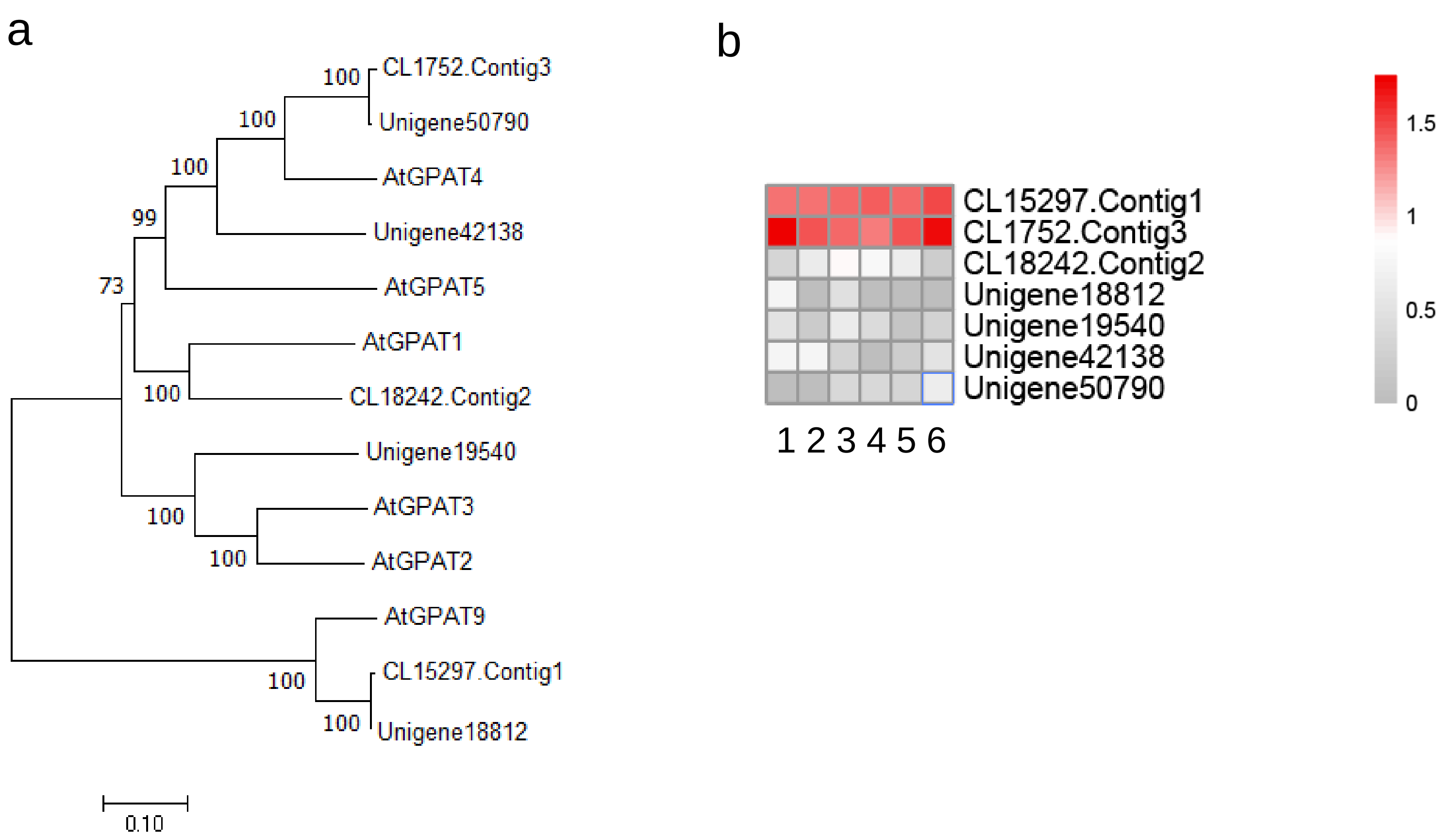

Supplemental Figure S7. Identification and analysis of GPAT from oil tea developing seeds
(**a**) Unrooted phylogenetic tree of oil tea (Camellia oleifera) for glycerol-3-phosphate acyltransferase (GPAT) with functionally characterized ones from other plants. The alignment was generated using ClustalW and the unrooted phylogram was constructed by the neighbor-joining method in MEGA7 software.
(**b**) Heatmap analysis of gene expression patterns of GPAT transcripts in oil tea seeds at various developmental stages. Data are from transcriptome of developing seeds, RStudio was used form making the heatmap.

LPAAT

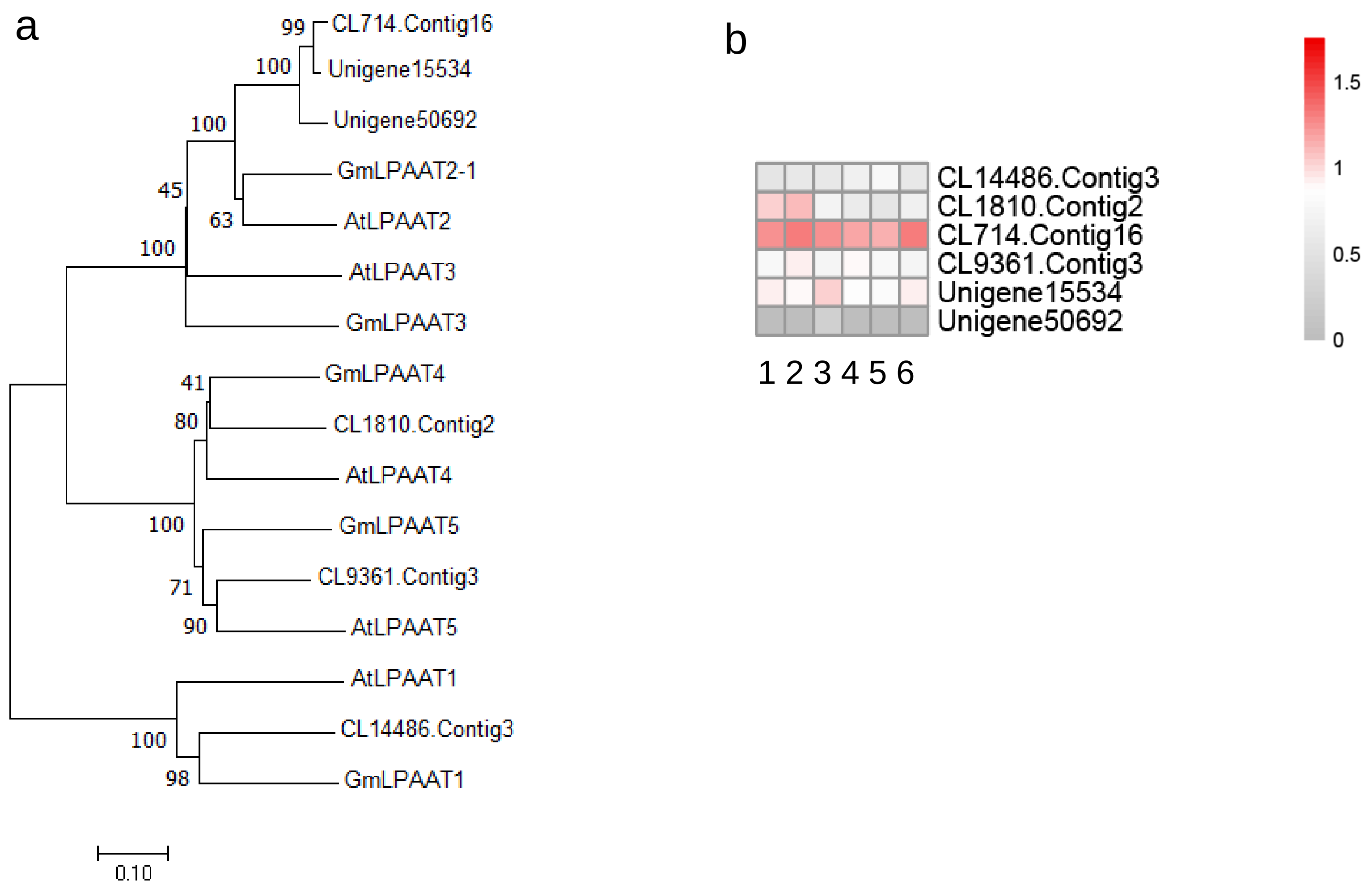

Supplemental Figure S8. Identification and analysis of LPAATs from oil tea developing seeds

(**a**) Unrooted phylogenetic tree of oil tea (Camellia oleifera) for acyl-CoA:LPA acyltransferase (LPAAT) with functionally characterized ones from other plants. The alignment was generated using ClustalW and the unrooted phylogram was constructed by the neighbor-joining method in MEGA7 software.

(**b**) Heatmap analysis of gene expression patterns of LPAAT transcripts in oil tea seeds at various developmental stages.

Data are from transcriptome of developing seeds, RStudio was used form making the heatmap.

PLA

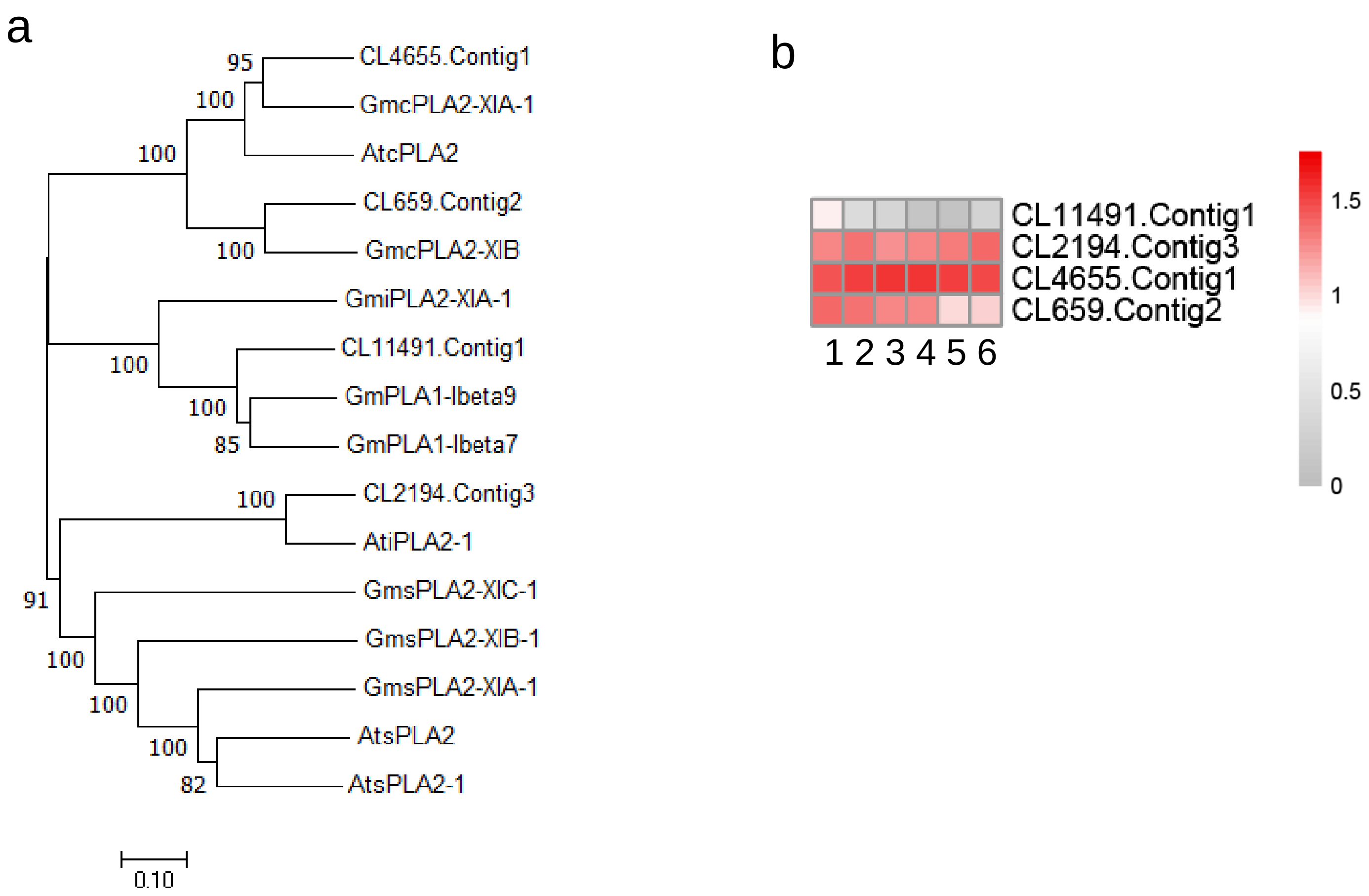

Supplemental Figure S9. Identification and analysis of PLAs from oil tea developing seeds

(**a**) Unrooted phylogenetic tree of oil tea (*Camellia oleifera*) for phospholipase A (PLA) with functionally characterized ones from other plants. The alignment was generated using ClustalW and the unrooted phylogram was constructed by the neighbor-joining method in MEGA7 software.

(**b**) Heatmap analysis of gene expression patterns of PLA transcripts in oil tea seeds at various developmental stages. Data are from transcriptome of developing seeds, RStudio was used form making the heatmap.

PLD

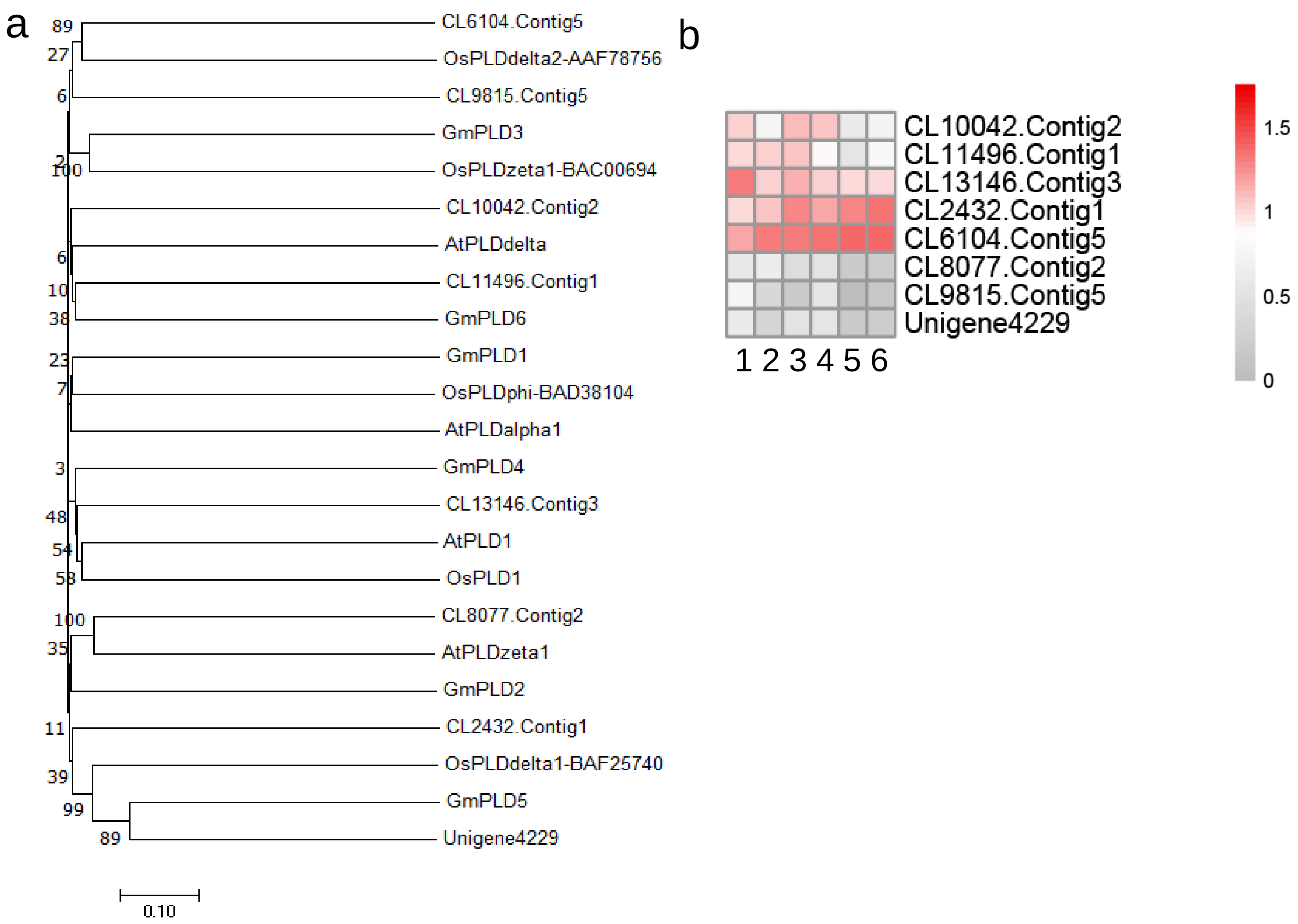

Supplemental Figure S10. Identification and analysis of PLDs from oil tea developing seeds
(**a**) Unrooted phylogenetic tree of oil Camellia (Camellia oleifera) for phospholipase D (PLD) with functionally characterized ones from other plants. The alignment was generated using ClustalW and the unrooted phylogram was constructed by the neighbor-joining method in MEGA7 software.
(**b**) Heatmap analysis of gene expression patterns of PLD transcripts in oil tea seeds at various developmental stages.
Data are from transcriptome of developing seeds, RStudio was used form making the heatmap.

PDAT

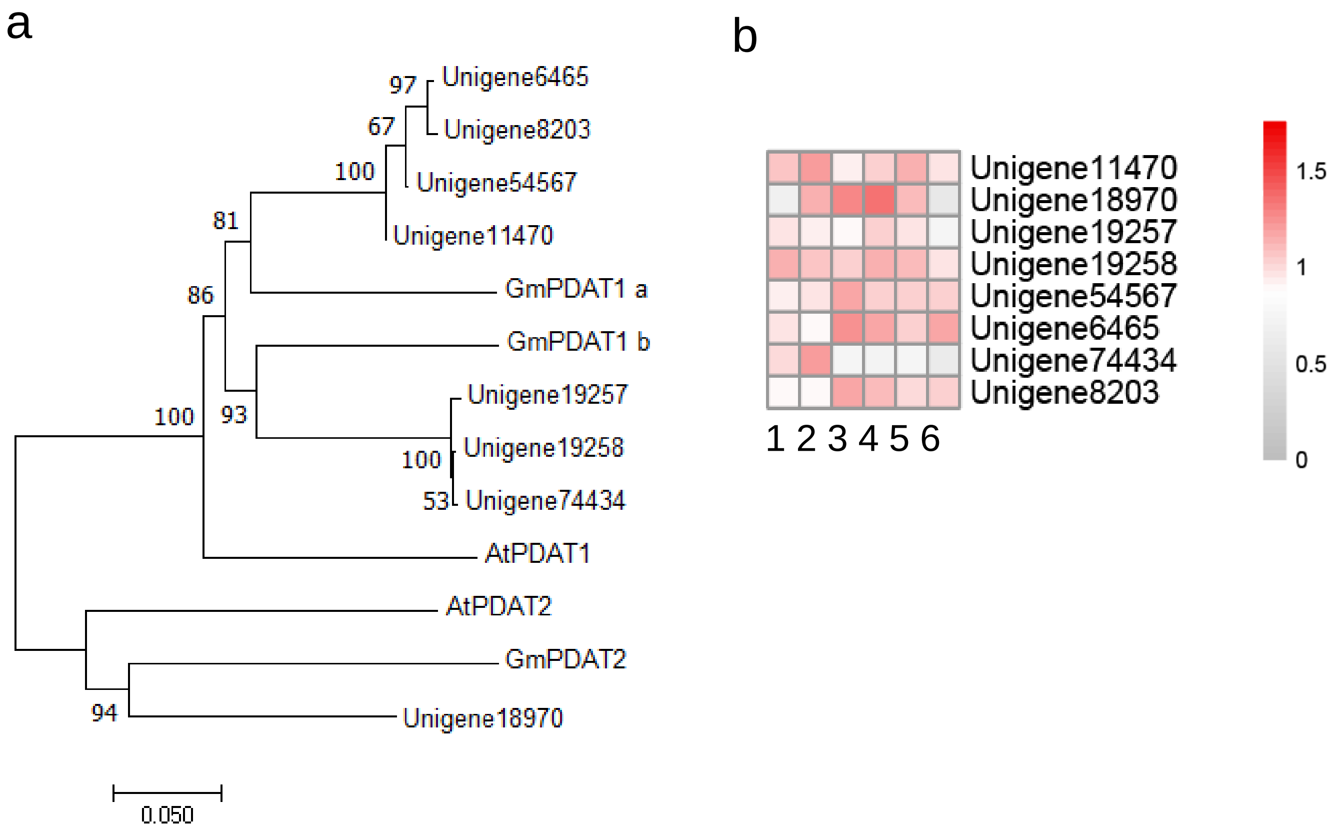

Supplemental Figure S11. Identification and analysis of PDATs from oil tea developing seeds

(**a**) Unrooted phylogenetic tree of oil tea (*Camellia oleifera*) for phospholipid:diacylglycerol acyltransferase (PDAT) with functionally characterized ones from other plants. The alignment was generated using ClustalW and the unrooted phylogram was constructed by the neighbor-joining method in MEGA7 software.

(**b**) Heatmap analysis of gene expression patterns of PDAT transcripts in oil tea seeds at various developmental stages. Data are from transcriptome of developing seeds, RStudio was used form making the heatmap.

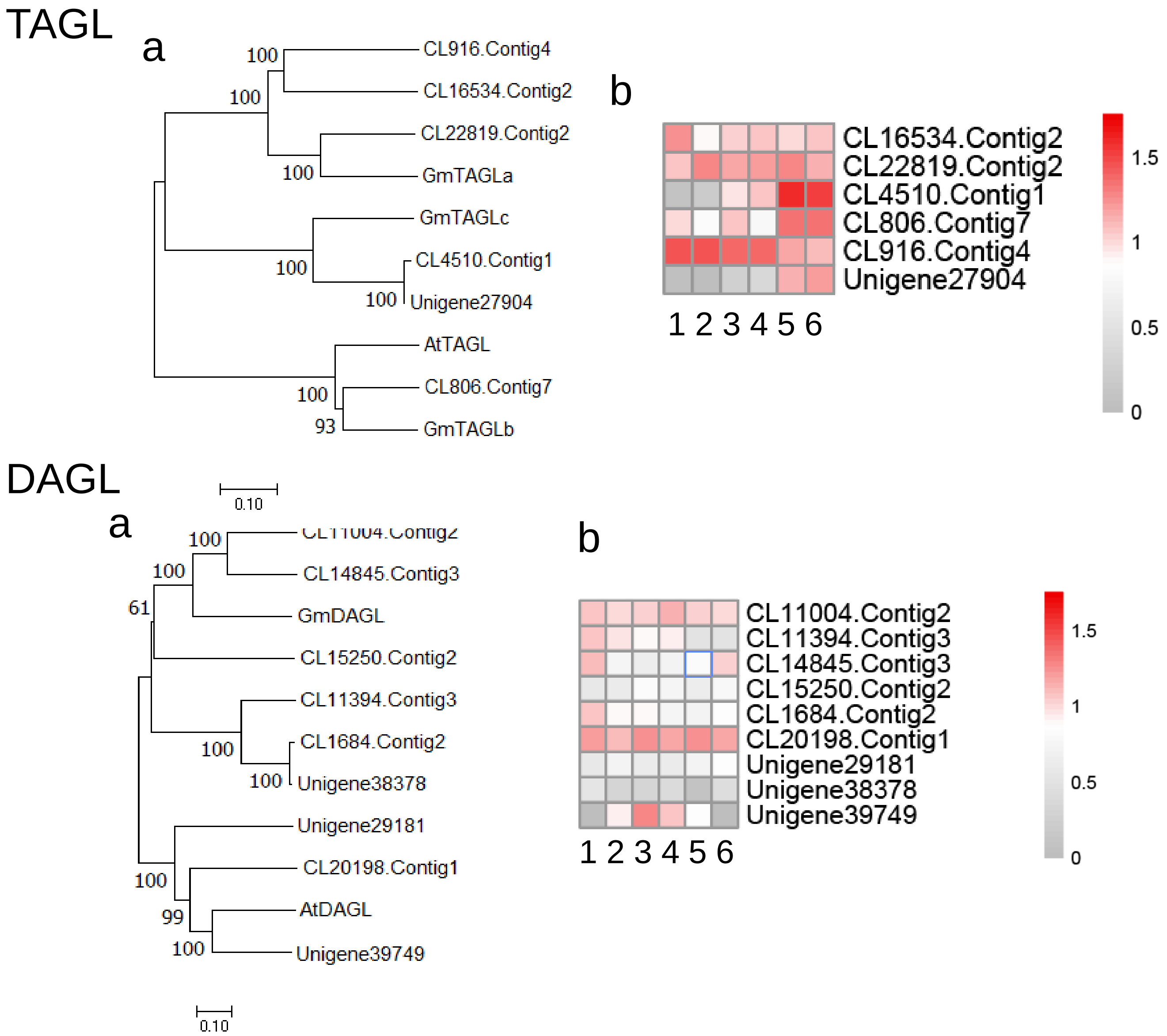

Supplemental Figure S12. Identification and analysis of TAGLs and DAGLs from oil tea developing seeds

(**a**) Unrooted phylogenetic tree of oil tea (Camellia oleifera) for Triacylglycerol Lipase (TAGL) and Diacylglycerol Lipase (DAGL) with functionally characterized ones from other plants. The alignment was generated using ClustalW and the unrooted phylogram was constructed by the neighbor-joining method in MEGA7 software.

(**b**) Heatmap analysis of gene expression patterns of TAGL and DAGL transcripts in oil tea seeds at various developmental stages.

Data are from transcriptome of developing seeds, RStudio was used form making the heatmap.

MAGL

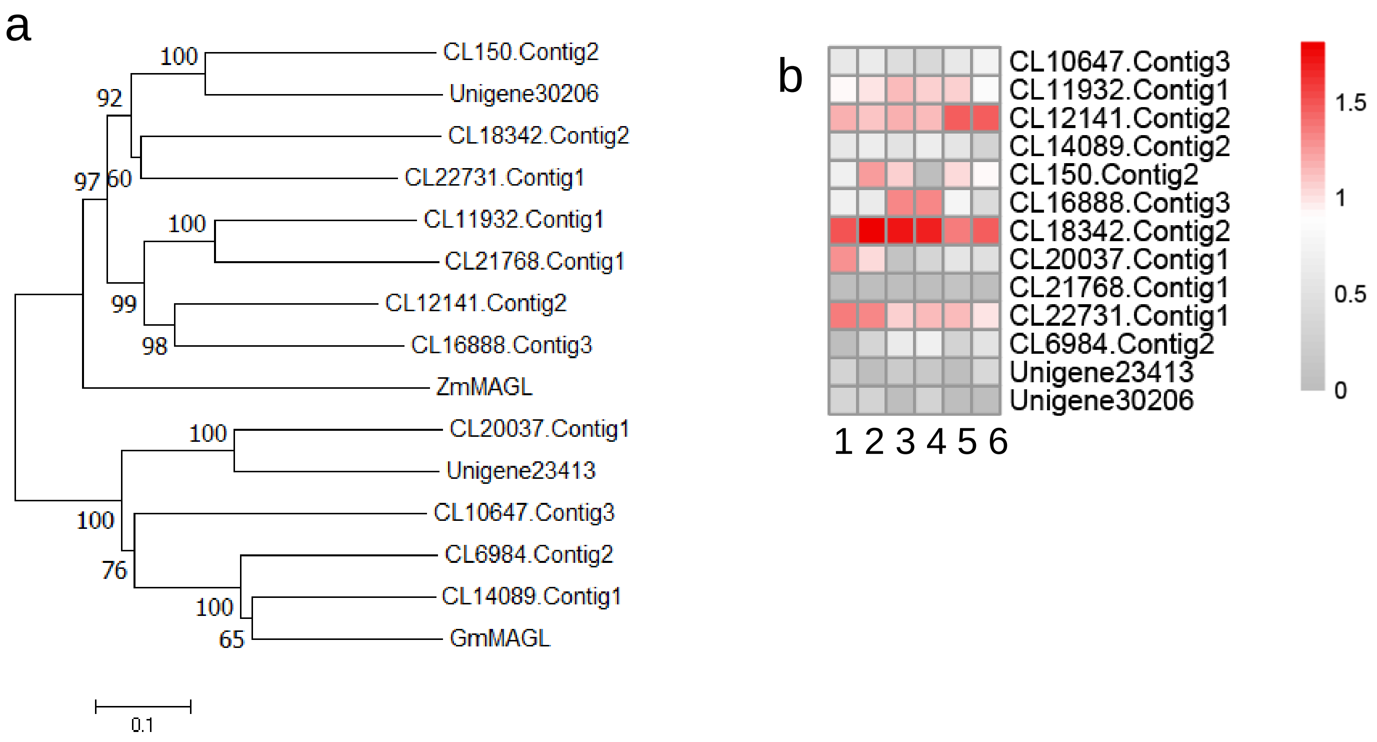

Supplemental Figure S13. Identification and analysis of MAGLs from oil tea developing seeds
(**a**) Unrooted phylogenetic tree of oil tea (*Camellia oleifera*) for Monoacylglycerol Lipase (MAGL) with functionally characterized ones from other plants. The alignment was generated using ClustalW and the unrooted phylogram was constructed by the neighbor-joining method in MEGA7 software.
(**b**) Heatmap analysis of gene expression patterns of MAGL transcripts in oil tea seeds at various developmental stages. Data are from transcriptome of developing seeds, RStudio was used form making the heatmap.

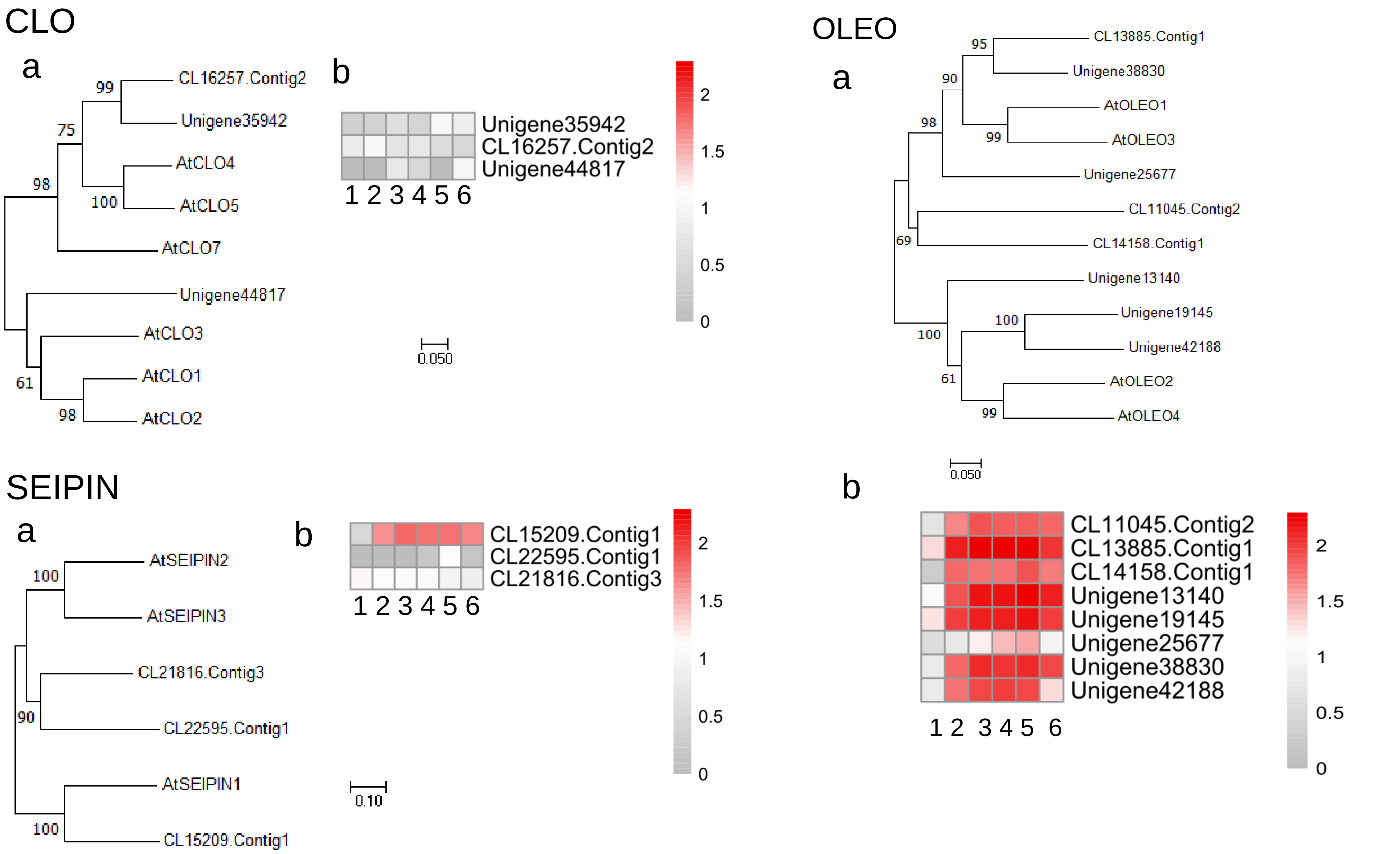

Supplemental Figure S14. Identification and analysis of CLO, SEIPIN and OLEO from oil tea developing seeds

(**a**) Unrooted phylogenetic tree of oil tea (*Camellia oleifera*) for caleosins (CLO), putative adipose-regulatory protein (SEIPIN) and oleosins (OLEO) with functionally characterized ones from other plants. The alignment was generated using ClustalW and the unrooted phylogram was constructed by t neighbor-joining method in MEGA7 software.

(**b**) Heatmap analysis of gene expression patterns of CLO, SEIPIN and OLEO transcripts in oil tea seeds at various developmental stages. Data are from transcriptome of developing seeds, RStudio was used form making the heatmap.

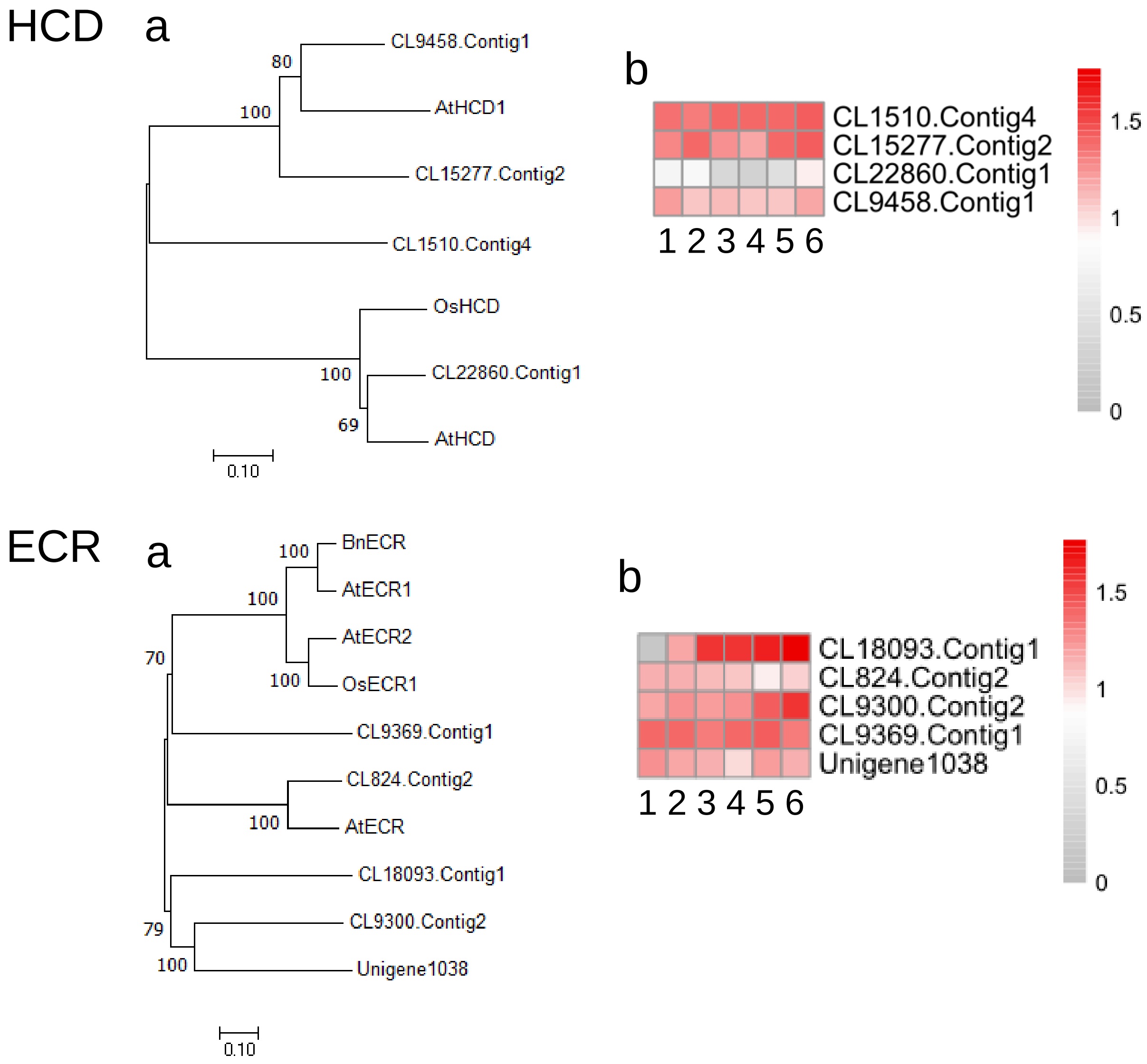

Supplemental Figure S15. Identification and analysis of HCD and ECR from oil tea developing seeds

(**a**) Unrooted phylogenetic tree of oil Camellia (Camellia oleifera) for β-hydroxyacyl-coA dehydratase (HCD) and enoyl-coA reductase (ECR) with functionally characterized ones from other plants. The alignment was generated using ClustalW and the unrooted phylogram was constructed by the neighbor-joining method in MEGA7 software.

(**b**) Heatmap analysis of gene expression patterns of HCD and ECR transcripts in oil tea seeds at various developmental stages. Data are from transcriptome of developing seeds, RStudio was used form making the heatmap.

Wax ester synthase/diacylglycerol acyltrasferase (WSD)

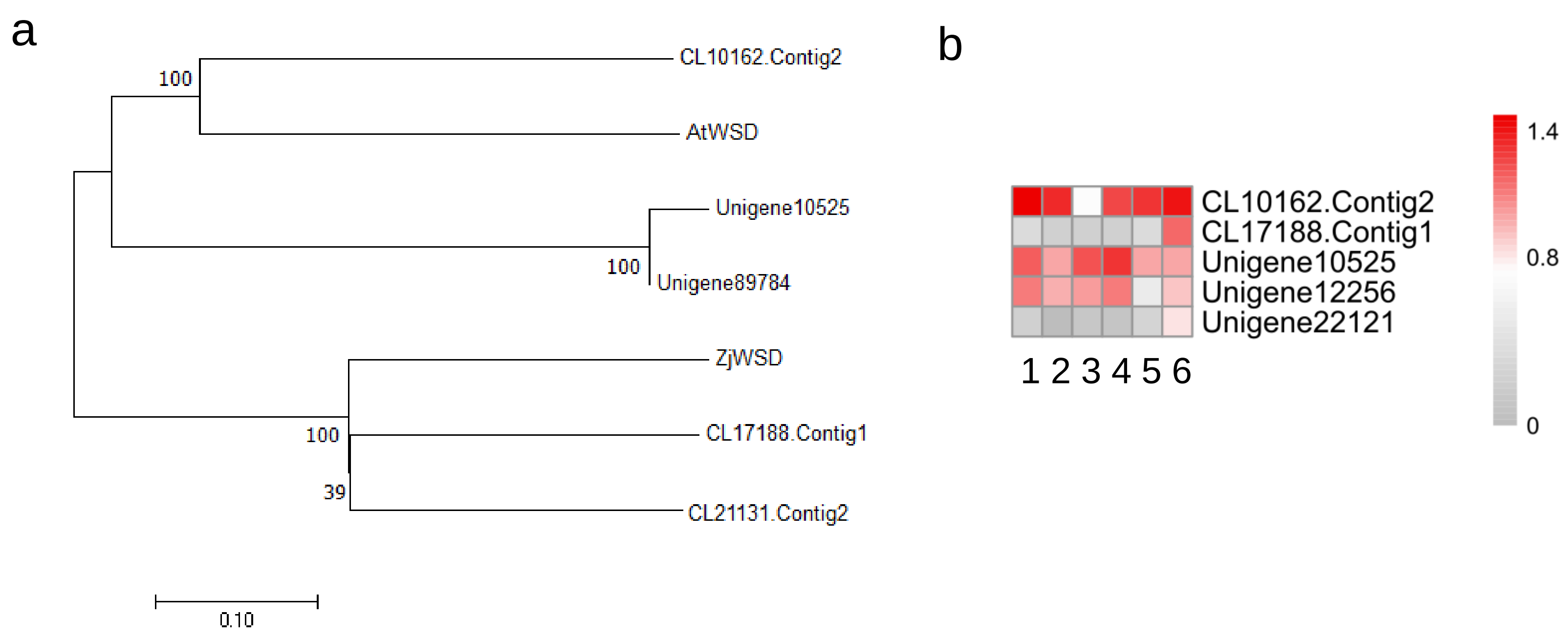

Supplemental Figure S16. Identification and analysis of WSDs from oil tea developing seeds

(**a**) Unrooted phylogenetic tree for Wax ester synthase/diacylglycerol acyltrasferase (WSD) with other functionally characterized WSDs. The alignment was generated using ClustalW and the unrooted phylogram was constructed by the neighbor-joining method in MEGA7 software.

(b) Heatmap analysis of gene expression patterns of WSD transcripts in developing oil tea seeds. Data are from transcriptome of developing seeds, RStudio was used form making the heatmap.

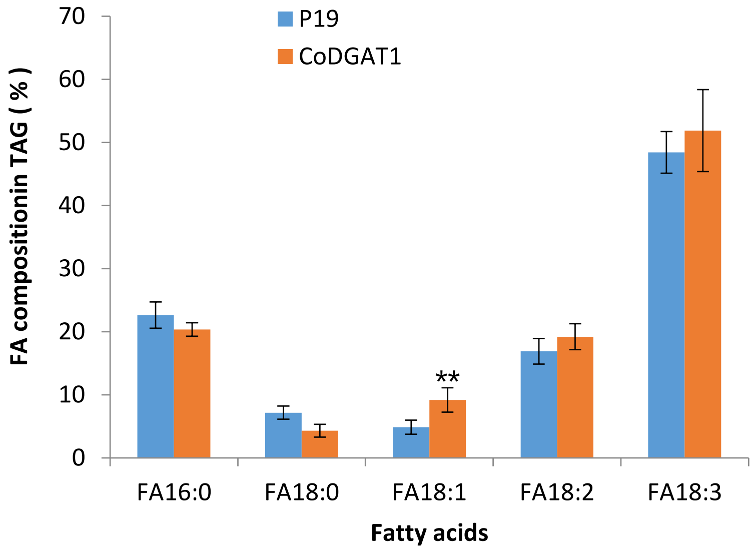

Supplemental Figure S17. Effects of *CoDGAT1* overexpression on TAG fatty acid compositions in tobacco leaf.

CoDGAT1 overexpression in tobacco leaf was driven by a cauliflower mosaic virus 35S promoter, and infiltrated tobacco leaves were harvested at 4-5 days after infiltration, as compared with that in tobacco leaves expressing empty vector P19. TAGs were extracted and separated by thin-layer chromatography. TAG spots were scraped off from the TLC plates and extracted for analysis of TAG contents and compositions with GC. All data are presented by means of at least three biological replicates $\pm$ SD. *$P < 0.05$ and **$P < 0.01$ by Student's *t* test for significant difference.

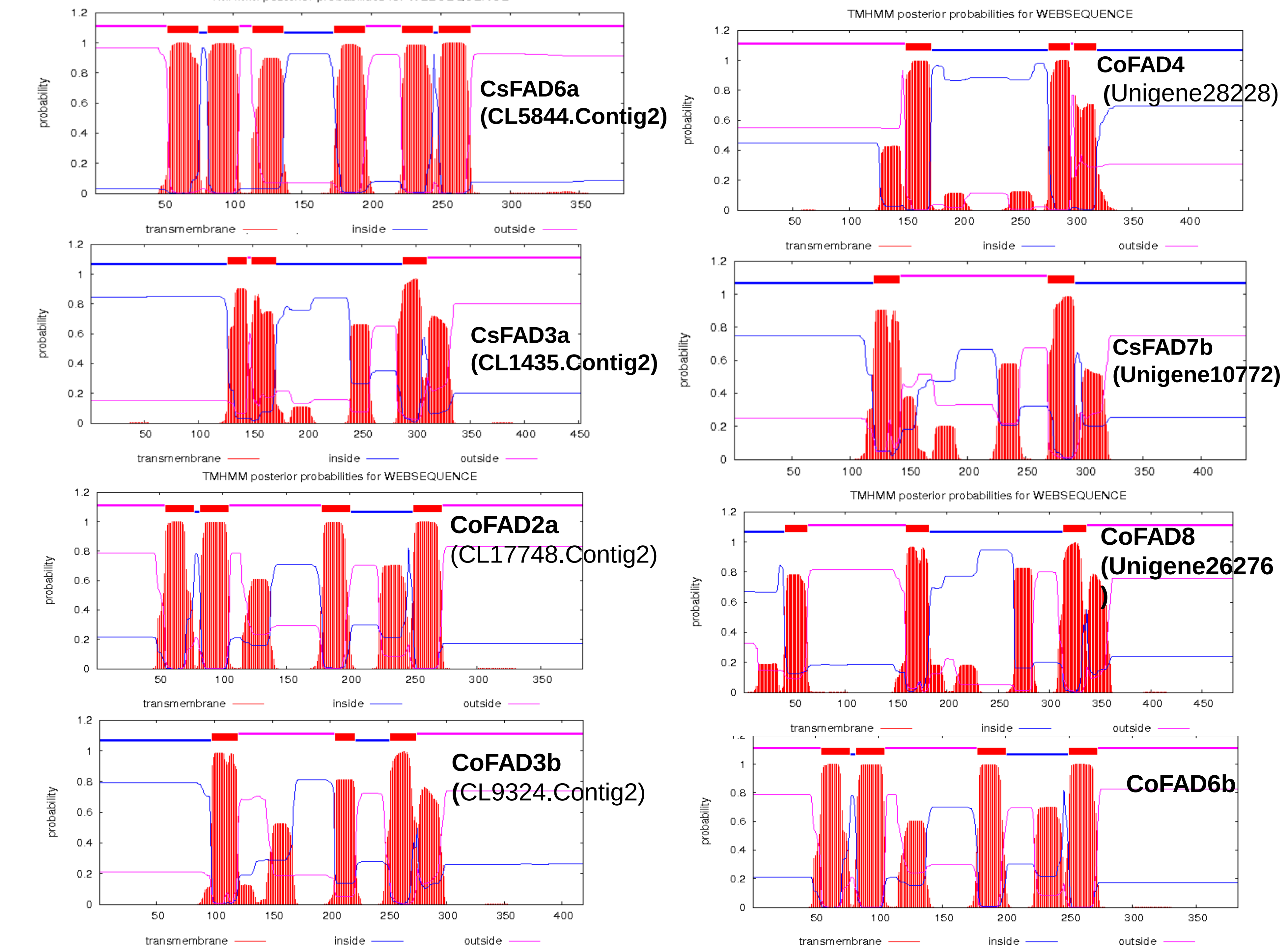
CsFAD6a
(CL5844.Contig2)
CoFAD4
(Unigene28228)
CsFAD3a
(CL1435.Contig2)
CsFAD7b
(Unigene10772)
CoFAD2a
(CL17748.Contig2)
CoFAD8
(Unigene26276
)
CoFAD3b
(CL9324.Contig2)
CoFAD6b
TMHMM posterior probabilities for WEBSEQUENCE
probability
transmembrane
inside
outside

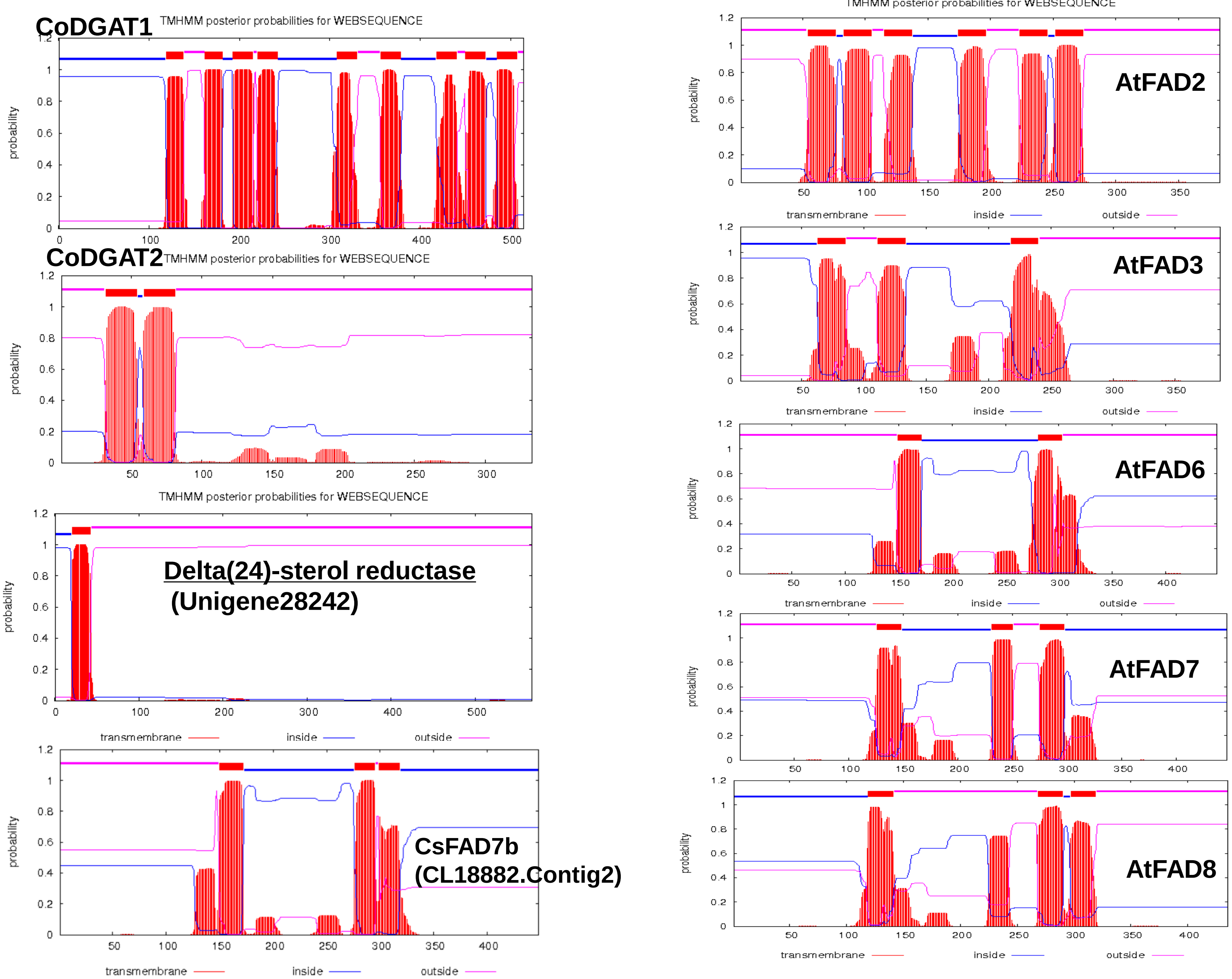

CoDGAT1
TMHMM posterior probabilities for WEBSEQUENCE
CoDGAT2
TMHMM posterior probabilities for WEBSEQUENCE
TMHMM posterior probabilities for WEBSEQUENCE
Delta(24)-sterol reductase
(Unigene28242)
CsFAD7b
(CL18882.Contig2)
TMHMM posterior probabilities for WEBSEQUENCE
AtFAD2
AtFAD3
AtFAD6
AtFAD7
AtFAD8
probability
transmembrane
inside
outside

Supplemental Figure S18. Topological analysis of CoFAD, CoSAD, CoDGATs from oil tea plants, as compared with FADs from Arabidopsis.

The transmembrane domains (TMs) underlined were predicted with the TMHMM Server ver. 2.0 (http://www.cbs.dtu.dk/ services/TMHMM/).

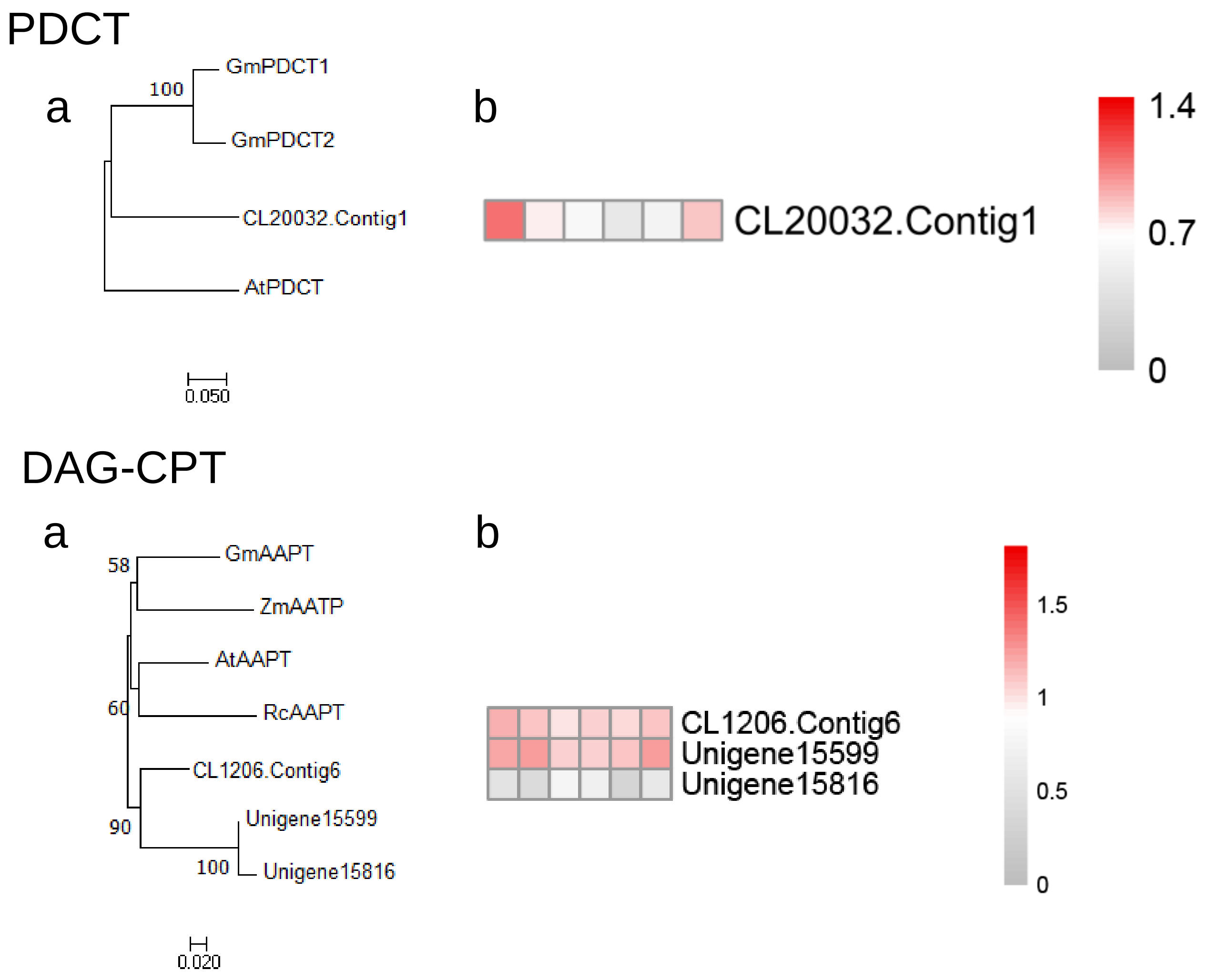

Supplemental Figure S19. Bioinformatics analysis of PDCTs and DAG-CPTs from oil tea and other plants
(**a**) Unrooted phylogenetic tree of oil tea (*Camellia oleifera*) for PDCT (Phosphatidylcholine: diacylglycerol cholinephosphotransferase) and DAG: cholinephosphotransferase (DAG-CPT) with functionally characterized ones from other plants. The alignment was generated using ClustalW and the unrooted phylogram was constructed by the neighbor-joining method in MEGA7 software.
(**b**) Heatmap analysis of gene expression patterns of PDCT and DAG-CPT transcripts in oil Camellia seeds at various developmental stages. Data are from transcriptome of developing oil tea seeds, RStudio was used form making the heatmap.
