# Supplementary material for: Critical metabolic pathways and SAD/FADs, WRI1s, and DGATs cooperate for high-oleic acid oil production in developing oil tea (Camellia oleifera) seeds
Source: Hortic Res. 2022 Apr 21;9:uhac087. doi: 10.1093/hr/uhac087 (PMC9178347; doi:10.1093/hr/uhac087)
Supplement: Web_Material_uhac087 [file web_material_uhac087.zip › Supplemental Dataset S1-S3 Glycolysis and lipid synthesis gene expression patterns.pdf]

Supplemental Dataset S1 **Supplemental Dataset S1 Glycolysis gene expression patterns**

Seed developmental Stages		1	2	3	4	5	6
**STS (starch synthase)**	CL4411.Contig1	7.56	8.51	7.19	7.84	6.78	5.27
	CL16745.Contig2	25.67	72.57	50.45	76.85	10.94	1.48
	CL12760.Contig1	13.95	9.68	20.84	14.46	18.88	17.51
**SUS (sucrose synthase)**	Unigene28501	5.62	2.31	1.81	2.11	19.68	9.91
	Unigene41968	3.75	198.63	331.49	351.64	165.51	4.21
	CL19004.Contig1	110.1	182.81	23.46	17.62	2.49	1.71
	CL6660.Contig4	3.3	1.62	2.39	2.08	1.93	2.24
**INVs (invertase)**	CL170.Contig4	26.32	15.43	58.88	66.73	75.32	183.3
	CL7533.Contig1	0.13	0	0.35	0.16	0	0
	CL16442.Contig3	3.87	5.84	2.07	1.45	0.31	0.45
	CL3811.Contig3	3.52	3.89	2.45	2.17	0.95	1.94
	CL3327.Contig2	9.45	7.09	8.04	8.69	6.64	0
	CL19058.Contig1	6.26	5.11	1.76	1.84	6.36	4.53
**Amy (Amylase)**	CL2998.Contig4	19.94	1.97	6.39	4.08	7.86	1.02
	CL3471.Contig3	4.85	9.54	13.88	18.51	10.6	10.09
	CL2880.Contig2	6.21	4.2	6.66	11.89	6.2	2.88
	CL6714.Contig5	9.9	7.79	9.78	11.66	6.62	6.99
	Unigene41746	8.86	7.73	10.35	14.96	6.69	6.69
	CL3366.Contig4	4.25	5.35	3.8	2.83	4.66	7.71
	CL3151.Contig4	27.82	25.38	135.36	79.62	218.6	143.7
	Unigene26038	3.34	4.14	3.62	6.05	6.05	10.27
	Unigene39349	3.12	3.83	7.89	10.03	3.4	3.41
	Unigene55105	14.27	27.66	16.9	16.21	9.99	4.09
	CL22101.Contig3	6.66	8.29	6.53	4.9	4.26	1.41
	CL13510.Contig2	10.47	4.03	0.74	0.58	0.36	0.36
	CL12963.Contig1	6.27	5.92	5.24	6.27	1.76	1.85
	CL15523.Contig2	8.76	10.67	8.54	9.98	8.2	13.42
**HXK (hexokinase)**	Unigene28745	13.65	17.18	25.68	26.53	23.86	24.56
	Unigene45777	16.63	21.78	36.23	38.68	34.89	42.86
	CL20299.Contig2	17.77	18.98	17.19	20.23	5.3	1.87
**PGI (glucose phosphate**	CL2448.Contig5	1.37	1.07	6.91	6.23	9.25	7.27

**isomerase)**							
	Unigene32265	7.2	7.22	10.28	7.51	16.72	8.66
**PPi-PFK (phosphofructokinase)**	CL14453.Contig3	2.32	3.61	3.02	2.9	2.03	1.32
	CL16016.Contig3	13.05	8.77	10.77	10.83	21.25	21.67
	CL2390.Contig1	38.75	40.43	22.41	21.76	9.81	7.85
	CL3389.Contig4	11.18	19.68	31.14	20.6	11.34	26.06
	Unigene18642	4.62	7.57	12.52	7.84	5.62	11.66
	Unigene18644	20.63	33.89	7.79	7.98	1.8	3.9
	Unigene19179	1.04	4.08	18.23	15.58	11.79	4.11
	Unigene19180	2.31	13.29	15.52	24.13	19.7	5.75
	Unigene28929	7.04	14.17	10.42	12.92	10.64	7.51
	Unigene38615	43.62	73.99	49.47	50.92	68.55	30.51
**ALDO (fructose-bisphosphate aldolase)**	CL11503.Contig3	36.57	164.64	112.54	146.15	62.05	60.78
	CL17862.Contig1	223	366.25	275.75	284.97	131.8	183.8
	Unigene16142	7.49	13.07	9.36	9.68	2.11	2.07
**G3PDH (glyceraldehyde-3-phosphate dehydrogenase)**	CL13930.Contig1	4.09	4.36	2.02	0.87	2.89	9.78
	CL22004.Contig2	10.1	42.18	7.36	15.76	8.47	5.68
	CL5014.Contig5	37.09	109.38	91.93	121.7	52.18	40.47
	CL7038.Contig3	165.9	191.29	151.51	116.93	35.77	27.06
	Unigene14271	518.3	817.47	674.79	777.47	895.85	586.1
	Unigene16136	613.5	1013.19	769.79	909.12	1058.3	756.6
	Unigene86028	3.94	12.71	1.08	7.89	3.15	2.36
**PGK (phosphoglycerate kinase)**	CL6944.Contig3	10.44	14.29	10.92	12.65	4.7	7.64
	Unigene17852	166.4	323.35	159.68	188.29	64.14	68.73
**PGM (2,3-bisphosphoglycerate-independent phosphoglycerate mutase)**	CL12434.Contig3	69.42	79.67	45.84	42.86	36.53	50.99

	CL13181.Contig2	0.9	6.3	0.58	3.29	0.27	0.13
	Unigene38531	14.17	13.3	48.6	23.92	5.57	4.99
**ENO (enolase)**	CL10509.Contig2	14.36	42.15	21.5	22.6	8.91	2.95
	CL10757.Contig1	147.3	419.42	242.37	362.75	154.9	78.12
	Unigene14788	147.4	121.11	28.13	17.92	5.8	11.72
	Unigene8667	169.3	125.18	32.11	18.55	4.91	11.96
**PK (pyruvate kinase)**	CL2850.Contig3	16.21	20.26	18.25	17.4	5.15	5.65
	CL9219.Contig3	126.9	199.9	100.76	125.65	58.37	75.3
	Unigene13699	30.34	32.21	13.93	11.17	4.73	11.33
	Unigene14495	28.11	29.54	13.85	10.65	5.1	9.94

Supplemental Dataset S2 Lipid biosynthetic gene expression patterns

**Seed developmental Stages**		**1**	**2**	**3**	**4**	**5**	**6**
**ACCase (Acetyl-CoA carboxylase)**	CL1061.Contig4	16.47	50.06	50.12	68.39	15.2	9.55
	CL2005.Contig8	2.62	2.11	4.73	4.31	6.71	3.51
	CL4377.Contig2	2.96	8.42	6.88	7.84	2.99	1.24
	CL5954.Contig3	13.21	27.01	37.8	27.12	46.6	54.64
	Unigene12477	1.1	2.47	4.26	3.26	0.61	0.34
	Unigene16476	0.96	4.08	3.87	6.21	3.78	1.6
	Unigene17024	4.24	11.46	16.54	20.32	7.85	4.9
	Unigene17580	1.06	3.76	2.61	4.16	0.44	0.31
	Unigene6929	8.96	19.11	33.19	42.41	19.5	10.98
	Unigene72627	3.22	9.43	14.24	17.79	10.1	6.02
**MAT (malonyl-CoA-ACP transacylase)**	CL6219.Contig1	12.49	35.87	24.18	28.37	5.4	4.28
**KASIII (3-oxoacyl ACP synthase)**	CL1668.Contig2	4.09	14.78	11.78	22.24	15.1	6.12
	Unigene25219	6.27	8.49	7.35	8.37	3.56	3.83
	Unigene25220	5.76	10.55	9.64	8.97	3.6	3.06
**KAR (3-oxoacyl ACP reductase)**	CL2489.Contig1	8.07	20.74	25.09	29.39	6.17	3.14
	Unigene5786	0.64	2.34	2.41	3.14	0.6	0.88
	Unigene7741	0.15	0.81	2.01	3.15	0.13	0.86
**KASII (3-oxoacyl ACP synthase)**	CL1914.Contig2	33.84	50.36	77.95	79.82	26.2	20.77

**KASI (3-oxoacyl ACP synthase)**	CL7620.Contig2	17.15	40.82	77.9	94.05	34.1	21.85
**SAD (stearoyl-ACP desaturase)**	CL17700.Contig2	14.83	27.55	18.27	32.46	4.72	2.05
	CL23116.Contig1	12.21	147.22	184.5	226.33	66.9	12.8
	CL9407.Contig1	43.61	39.14	551	472.15	10.6	23.26
**FAT(A,B) (fatty acyl-ACP thioesterase A/B)**	CL53.Contig4	31.77	35.63	29.61	28.37	22.4	15.33
	CL7284.Contig2	4.35	8.97	15.36	20.84	16.9	17.74
	Unigene16664	17.75	24.18	12.47	12.09	8.26	6.54
**TGDs (Trigalactosyl diacylglycerol)**	CL10344.Contig1	15.45	27.23	21.93	23.72	15.6	8.46
	CL10412.Contig5	7.57	5.5	6.61	7.01	2.73	6.25
	CL16594.Contig1	35.21	72.14	70.15	69.63	119	84.86
	CL1790.Contig4	4.01	11.28	11.45	10.58	4.03	12.94
	CL18632.Contig1	11.58	9.78	6.43	6.55	3.7	4.08
	CL4935.Contig5	3.63	5.49	9.35	4.81	1.26	0
	CL814.Contig5	44.8	56.67	63.79	86.38	80.4	26.33
	Unigene22662	26.61	26.54	23.93	29.52	25.1	32.74
	Unigene45869	5.48	2.24	0.63	0.47	0.73	1.75
**FAX1 （Fatty acid export1 ）**	CL10344.Contig2	15.13	22.21	21.32	23.19	12	8.33
**LACS9 (long chain acyl-CoA synthetase)**	CL16130.Contig3	4.13	28.01	21.8	25.71	7.45	1.25
**HAD (3-hydroxyacyl-ACP dehydratase)**	Unigene12653	0.47	0.22	0.43	0.63	0.09	0.11
	Unigene7301	6.96	8.58	5.55	8.72	6.95	2.64
**EAR (enoyl-ACP reductase)**	Unigene72792	4.25	8.68	5.34	5.81	1.97	3.23
**GPAT (glycerol-3-phosphate acyltransferase)**	CL15297.Contig1	16.21	17.49	17.92	21.6	18.1	32.38
	CL1752.Contig3	119.9	28.11	20.18	14.07	27.8	91.04
	CL18242.Contig2	0.47	1.38	3.23	2.36	1.54	0.3
	Unigene18812	1.99	0	0.82	0	0	0
	Unigene19540	0.94	0.24	1.4	0.61	0.16	0.44
	Unigene42138	1.98	2.18	0.45	0	0.28	0.93
	Unigene50790	0	0	0.53	0.59	0.5	1.55

**LPAAT (acyl-CoA::LPA acyltransferase)**	CL14486.Contig3	1.05	1.15	1.14	1.73	2.23	1.24
	CL1810.Contig2	5.39	6.41	1.86	1.37	1.02	1.7
	CL714.Contig16	10.94	14.74	11.45	8.4	7.66	14.22
	CL9361.Contig3	2.23	3.71	2.16	3.11	2.33	2.11
	Unigene15534	3.84	3.25	5.25	2.95	2.64	3.53
	Unigene50692	0	0	0.37	0	0	0
**PAP (phosphatidic acid phosphatase)**	CL4504.Contig2	10.32	6.48	11.06	11.21	9.08	14.04
	CL6901.Contig5	4.78	5.3	7.23	4.02	2.38	2.06
**DGAT (diacylglycerol O-acyltransferase)**	CL1666.Contig2	12.12	9.48	13.42	10.28	3.84	5.48
	CL21487.Contig7	8.01	7.59	3.12	2.65	10.7	20.09
	Unigene22423	26.27	44.35	86.95	50.77	17.5	31.23
	Unigene7038	1.2	1.3	0.56	0.99	5.73	6.35
	Unigene7306	0	0	0.08	0	0.35	0.02
	Unigene18350	0	0	0.71	0	0.37	0.89
	Unigene7525	2.55	1.35	0.76	2.12	0.25	0.09
	Unigene42750	0.58	2.65	0.67	0.93	0.19	2.3
	Unigene14386	10.08	8.5	5.97	5.65	2.47	3.11
	CL17093.Contig2	0.37	0.24	0	0.35	0.18	0
**PDAT (phospholipid:diacylglycerol acyltransferase)**	Unigene11470	5.65	9.74	3.73	5	7.21	4.25
	Unigene18970	1.75	7.16	12.66	16.42	6.28	1.18
	Unigene19257	4.19	3.62	3.23	5.29	4.01	1.99
	Unigene19258	7.38	5.73	5.46	7.2	6.69	4.3
	Unigene54567	3.76	4.13	8.45	5.27	5.38	5.16
	Unigene6465	4.01	3.44	11.64	8.88	5.12	8.22
	Unigene74434	4.52	9.59	2.04	1.91	2.03	1.29
	Unigene8203	3.41	3.14	8.68	6.73	4.81	5.3
**TAGL (Triacylglycerol Lipase)**	CL16534.Contig2	11.12	3.39	5.27	6.14	4.51	6.1
	CL22819.Contig2	5.58	11.97	8.24	9.42	13.4	7.77
	CL4510.Contig1	0.1	0.22	4.03	5.51	54.9	37.16
	CL806.Contig7	4.8	2.66	5.65	2.44	15.7	15.76
	CL916.Contig4	26.83	24.98	20.33	18.17	8.46	6.75
	Unigene27904	0.05	0	0.37	0.57	7.75	9.43
**DAGL (Diacylglycerol Lipase)**	CL11004.Contig2	5.67	4.71	5.33	7.09	4.91	4.76
	CL11394.Contig3	5.87	3.98	3.3	3.51	0.97	0.91

	CL14845.Contig3	6.24	2.15	1.52	1.88	2.75	4.96
	CL15250.Contig2	1.15	1.39	2.69	2.07	1.45	2.45
	CL1684.Contig2	5.49	3.09	3.36	2.08	1.82	2.9
	CL20198.Contig1	9.79	6.77	10.4	8.46	10.4	8.15
	Unigene29181	1.26	1.87	1.41	1.28	1.82	2.79
	Unigene38378	1.02	0.48	0.49	0.62	0.1	0.76
	Unigene39749	0	3.8	13.11	6.04	2.88	0
**MAGL (Monoacylglycerol Lipase)**	CL10647.Contig3	1.34	1.47	0.74	0.62	1.27	1.97
	CL11932.Contig1	3.47	4.56	7.53	5.98	5.8	2.75
	CL12141.Contig2	8.28	6.9	8.61	7.75	29	26.16
	CL14089.Contig2	1.28	1.66	1.04	1.58	1.08	0.43
	CL150.Contig2	1.89	10.61	5.61	0	4.97	3.46
	CL16888.Contig3	1.88	1.45	14.46	14.07	2.2	0.66
	CL18342.Contig2	34.44	152.79	90.49	84.51	17.6	25.87
	CL20037.Contig1	13.39	5.42	0.09	0.53	1.17	0.84
	CL21768.Contig1	0	0	0	0	0.08	0
	CL22731.Contig1	17.46	15.64	5.47	7.82	7.73	4.43
	CL6984.Contig2	0	0.52	1.47	1.86	0.45	1.04
	Unigene23413	0.42	0	0.23	0.2	0	0.61
	Unigene30206	0.5	0.45	0	0.47	0	0
**PLA (phospholipase A)**	CL11491.Contig1	4	0.64	0.51	0.16	0.11	0.4
	CL2194.Contig3	13.77	19.35	12	13.86	17.8	22.57
	CL4655.Contig1	31.22	42.97	52.13	52.07	46.3	35.62
	CL659.Contig2	23.46	18.72	13.72	15.68	5.29	5.69
**FAD (fatty acid desaturase)**	CL1435.Contig3	24.68	39.55	11.17	11.15	1.34	0.36
	CL17748.Contig2	126.7	166.83	38.02	21.23	8.11	25.82
	CL5844.Contig2	15.33	23.1	113.8	95.81	95.7	92.43
	CL9324.Contig2	4.52	8.45	7.34	8.91	1.64	3.24
	Unigene10772	3.53	3.75	4.65	3.8	2.09	2.74
**PLD (phospholipase D）**	CL10042.Contig2	5.99	2.3	7.19	6.77	1.37	2.11
	CL11496.Contig1	5.23	5.55	6.24	3.7	1.06	2.46
	CL13146.Contig3	17	5.67	8.47	5.72	5.04	5.04
	CL2432.Contig1	5.14	6.7	15.5	10.15	15.3	19.27
	CL6104.Contig5	10.23	17.54	15.92	19.33	21.7	22.36
	CL8077.Contig2	1.34	1.63	0.75	0.96	0.22	0.32
	CL9815.Contig5	2.26	0.59	0.22	1.11	0	0.19
	Unigene4229	1.39	0.51	1.04	1.06	0.27	0.32
**OLEO(oleosin)**	Unigene25677_All	1.16	2.24	9.48	26.88	38.32	3.94

CL11045.Contig1_All	3.57	121.5	455.67	400.09	319.66	236.39
CL11045.Contig2_All	1.75	83.04	294.7	256.33	232.61	149.42
CL13872.Contig1_All	12.5	4.96	13.11	1.61	2.71	15
CL13885.Contig1_All	13.33	1581.49	6147.7	7077.81	6262.79	872.92
CL13885.Contig2_All	9.75	1369.32	5222.76	6212.69	5384.44	758.72
CL14158.Contig1_All	0.39	169.58	132.03	121.16	280.49	100.9
CL15500.Contig1_All	0	0	0	7.68	0	0
CL20713.Contig1_All	21.85	9.74	15.81	6.85	3.91	1.19
CL20713.Contig2_All	25.73	11.72	21.07	7.56	3.37	1.43
CL21477.Contig2_All	1.71	0	0	0	0	0.85
CL21725.Contig1_All	0	0	0.83	0	0	0
CL21725.Contig2_All	1.36	0	0	0	0	0.35
CL21961.Contig1_All	0.17	0.76	2.88	0.97	1.99	1.19
CL2341.Contig1_All	7.55	6.06	13.29	5.25	3.42	12.23
CL2341.Contig3_All	15.16	8.05	15.11	15.6	8.28	17.16
CL515.Contig12_All	0	0	0.14	0	0	0
CL6027.Contig1_All	0	0.19	0	0	0.07	0.09
CL6027.Contig2_All	2.26	3.13	5.2	5.41	0.53	1.79
CL6027.Contig3_All	2.71	2.32	2.3	0.63	0.57	0.23
CL6350.Contig2_All	6.09	33.15	75.99	125.81	174.69	112.85
CL9123.Contig2_All	1.59	1.81	1.02	1.32	1.08	1.46
Unigene10699_All	0.9	2.46	3.66	2.02	3.27	5.52
Unigene13099_All	6.24	5.52	5.66	0.42	12.37	0.84
Unigene13140_All	5.52	331.57	2597.74	2168.99	7530.39	1625.4
Unigene19145_All	10.65	603.56	1503.02	1586.6	2524.03	522.8
Unigene25677_All	1.16	2.24	9.48	26.88	38.32	3.94
Unigene2727_All	0.72	0	2.81	0.82	0	5.94
Unigene29281_All	5.34	6.79	7.37	4.57	7.19	5.93

	Unigene38829_All	0.38	68.53	65.72	323.73	608.61	232.81
	Unigene38830_All	2.34	157.39	1019.14	815.98	1148.71	413.29
	Unigene42188_All	2.58	139.12	386.19	578.27	372.29	15.16
	Unigene55837_All	0	0	0	0	0	1.35
	Unigene89333_All	0.79	0	1.91	0.82	0	0
**Cals(caleosin)**	Unigene35942_All	0.5	0.56	1.27	0.71	5.24	3.36
	CL16257.Contig2_All	2.35	5.26	1.88	2.11	1.23	0.9
	CL22860.Contig2_All	5.46	70.88	260.3	268.11	107	201.12
	Unigene44817_All	0	0	2.04	0.89	0	4.31
**SEIPIN(putative adipose-regulatory protein)**	CL15209.Contig1_All	0.85	61.7	153.5	115.18	116	78.86
	CL22595.Contig1_All	0	0	0	0.24	6.98	0.2
	CL21816.Contig3_All	8.08	7.23	6.42	5.65	3.65	3.25

Supplemental Dataset S3: PacBio full-length transcripts for oilbody proteins

GeneID	Oil tea	seed	development	stages			Pfam_annotation
	Co1	Co2	Co3	Co4	Co5	Co6	
Olesins (OLEs)							
Isoform51106	0	71.01	60.19	53.28	116.01	40.04	Oleosin
Isoform51105	0.33	177.75	130.97	116.07	255.77	113.47	Oleosin
Isoform51108	0.25	78.31	125.6	88.1	170.7	97.56	Oleosin
Isoform51841	0	23.59	48.74	35.95	117.06	44.47	--
Isoform51840	0.53	140.2	134.77	114.66	219.8	94.91	--
Isoform51966	0	1.26	75.41	213.56	9.85	0.4	Oleosin
Isoform50003	2.8	262.55	1570.77	2475.32	2155.53	644.35	Oleosin
Isoform50002	0	1.87	246.44	50.97	4.91	1.12	Oleosin
Isoform98547	6.65	490	3236.62	5196.63	1588.73	1070.2	Oleosin
Isoform51810	1.25	26.05	252.72	249.38	634.59	320.61	Oleosin
Isoform98548	1.61	80.1	891.7	651.44	406.08	39.62	Oleosin;

Isoform98549	5.34	112.95	1062.05	181.52	7185.23	995.74	Oleosin
Isoform98550	14.61	763.77	7175.43	6755.26	7704.36	2210.07	Oleosin
Isoform49875	1.52	85.29	475.01	483.86	1515.6	331.02	Oleosin
Isoform49874	0.36	29.28	42.27	12.39	1013.65	91.41	Oleosin
Isoform49873	0	52.55	463.92	7.79	1335.09	464.94	--
Isoform52418	0	0	79.16	0.27	69.8	0	Oleosin
Isoform52417	10.7	599.75	489.52	728.28	1839.65	353.06	Oleosin
Isoform98685	0	0	1.13	2.52	7.77	0.75	Oleosin
Isoform98686	9.87	408.98	1119.01	1043.24	1457.02	345	Oleosin
Isoform50328	0.95	71.49	340.91	244.76	155.85	88.51	Oleosin
Isoform50326	9.81	568.07	1662.71	2019.82	2727.82	583.88	Oleosin
Isoform50327	0	8.11	458.12	88.18	62.25	43.27	Oleosin
Isoform57046	0	39.22	4.88	10.32	87.2	38.22	Oleosin
Isoform57041	0	7.9	68.58	35.76	46.49	9.61	Oleosin
Isoform57040	0.76	71.75	113.29	226.5	197.32	106.19	Oleosin
Isoform55775	0.45	2.54	63.78	0.41	2.4	0.47	Oleosin
Isoform53965	1.6	76.32	807.53	426.92	46.59	117.58	Oleosin
Isoform53968	6.15	501.48	2302.35	2009.79	3521.74	1581.96	Oleosin
Isoform53967	0	0	145.38	25.12	108.02	0	--
Isoform54311	0	0	0	0	10.78	0	Oleosin
Isoform56131	0	4.14	237.34	29.92	67.32	30.39	--
Isoform57002	0	5.96	30.92	16.08	1.88	0	Oleosin
Isoform57003	4.3	339.91	1455.32	2402.24	2648.09	970.19	Oleosin
Isoform55761	12.26	1525.55	5183.08	7461.77	5412.19	936.75	Oleosin
Isoform55600	1.62	462.07	1210.99	2049.51	826.43	286.12	--
Isoform100362	6	1128.17	5059.39	4216.69	4830.96	589.12	Oleosin
Isoform55762	0	124.56	308.26	351.47	119.09	0.27	Oleosin
Isoform62297	1.04	24.9	96.14	69.87	55.22	80.47	Oleosin
Isoform62296	1.91	106.53	465.29	362.22	335.58	200.51	Oleosin
Isoform102153	0.99	59.63	156.69	175.23	124.45	65.28	Oleosin
Isoform66514	2.28	99.1	256.4	433.12	674.46	431.66	Oleosin
Isoform67480	0.99	24.8	119.04	222.21	137.45	113.72	Oleosin
Isoform69827	2.19	216.03	385.81	803.3	482.02	10.88	Oleosin
Isoform69828	2.93	154.28	698.11	736.77	523.71	25.11	Oleosin
Caleosins (CLOs)							
Isoform51551	2.49	277.04	706.52	1108.64	1462.11	919	Caleosin related protein
Isoform51550	0.38	78.62	285.83	103.55	193.48	339.16	Caleosin related protein
Isoform52514	0.94	0.82	0	0.22	135.05	0	Caleosin related protein
Isoform52509	0	0.53	0	1.51	1	1.28	Caleosin related protein
Isoform52424	0.9	331.62	126.98	98.66	404.83	532.89	Caleosin related protein
Isoform51549	0.19	11.12	8.14	26.02	17.99	15.66	Caleosin related protein
Isoform51553	0.9	153.02	424.78	323.62	726.33	454.9	Caleosin related protein
Isoform73188	0	0.15	0.49	0.41	0.57	1.8	Caleosin related protein; Caleosin related protein

Isoform73189	0.77	0.35	0.55	1.2	0.83	0.8	Caleosin related protein
Isoform73190	0.28	0.82	0.97	0.55	3.39	1.62	Caleosin related protein
Isoform108557	0.79	1.99	2.05	1.48	11.9	9.71	EF hand;; EF-hand domain pair;; EF-hand domain;; EF
**Steroleosin (SLOs)**							
Isoform47314	1.32	36.57	96.44	56.63	121.05	435.14	Steroleosin, beta-hydroxysteroid dehydrogenase-like
Isoform47310	1.29	40.79	133.3	214.08	426.37	438.4	Steroleosin, beta-hydroxysteroid dehydrogenase-like
Isoform44486	1.07	39.82	215.52	275.97	570.8	480.76	Steroleosin, beta-hydroxysteroid dehydrogenase-like
Isoform47313	0.24	16.68	52.4	83.4	39.4	284.11	Steroleosin, beta-hydroxysteroid dehydrogenase-like
**SEIPINs**							
Isoform53505	0.16	1.16	24.3	5.77	3.48	7.54	Putative adipose-regulatory protein (Seipin)
Isoform53506	0	16.61	50.6	94.44	24.83	9.33	Putative adipose-regulatory protein (Seipin)
Isoform53504	1.21	92.12	189.77	171.47	160.77	117.26	Putative adipose-regulatory protein (Seipin)
Isoform106022	0.53	14.38	16.71	8.35	17.87	8.92	Putative adipose-regulatory protein (Seipin)
Isoform106023	0	8.17	4.05	4.65	6.29	3.78	Putative adipose-regulatory protein (Seipin)
Isoform35954	8.65	9.96	7.5	6.77	3.08	5.6	Putative adipose-regulatory protein (Seipin)
Isoform35953	7.82	4.27	3.34	3.09	2.03	2.62	Putative adipose-regulatory protein (Seipin)
